# Comprehensive Analysis of Circular RNA Expression in ceRNA Networks and Identification of the Effects of hsa_circ_0006867 in Keloid Dermal Fibroblasts

**DOI:** 10.3389/fmolb.2022.800122

**Published:** 2022-01-31

**Authors:** Qianqian Pang, Xiaohu Lin, Jiaqi Sun, Jie Hu, Siya Dai, Yichen Shen, Mingyuan Xu, Jinghong Xu

**Affiliations:** Department of Plastic Surgery, The First Affiliated Hospital, School of Medicine, Zhejiang University, Hangzhou, China

**Keywords:** circular RNA, keloid, dermal fibroblast, microarray analysis, ceRNA network, miRNA sponge

## Abstract

Circular RNAs (circRNAs) play a crucial role in the pathogenesis of various fibrotic diseases, but the potential biological function and expression profile of circRNAs in keloids remain unknown. Herein, microarray technology was applied to detect circRNA expression in four patient-derived keloid dermal fibroblasts (KDFs) and normal dermal fibroblasts (NDFs). A total of 327 differentially expressed (DE) circRNAs (fold change > 1.5, *p* < 0.05) were identified with 195 upregulated and 132 downregulated circRNAs. Gene Ontology (GO) and Kyoto Encyclopedia of Genes and Genomes (KEGG) pathway analyses showed that the upregulated circRNAs were mainly enriched in the cytoskeleton and tight junctions, while the downregulated circRNAs were related to morphogenesis of the epithelium and axonal guidance. To explore the function of DE circRNAs, a circRNA-miRNA-mRNA network, including five circRNAs, nine miRNAs, and 235 correlated mRNAs, was constructed using bioinformatics analyses. The expression of five DE circRNAs was validated by qRT–PCR in 18 pairs of KDFs and NDFs, and hsa_circ_0006867 showed promising regulatory function in keloids *in vitro*. Silencing hsa_circ_00006867 suppressed the proliferation, migration, and invasion of keloid fibroblasts. RNA-binding protein immunoprecipitation (RIP) assays indicated that hsa_circ_00006867 may serve as a platform for miRNA binding to Argonaute (AGO) 2. In addition, hsa-miR-29a-5p may be a potential target miRNA of hsa_circ_00006867. Taken together, our research provided multiple novel clues to understand the pathophysiologic mechanism of keloids and identified hsa_circ_0006867 as a biomarker of keloids.

## Introduction

Keloids are benign skin fibroproliferative tumors unique to humans characterized by aggressive fibroblast proliferation, excessive accumulation of extracellular matrix (ECM) (e.g., collagen I and III), long-term continuous fibrosis, and abnormal inflammatory stimuli (Yan et al., 2021; Zhang et al., 2021). Keloids show various cancer-like features, such as aggressive proliferation beyond the original boundary, lack of spontaneous regression, and vascularization ability (Huang and Ogawa, 2020). As a common clinically challenging disorder, keloid formation generally follows wounding, such as burns, surgery, laceration, or other damage to the dermis (Davies et al., 2021). Keloids are more likely to occur in areas with high skin tension, such as the chest, earlobes, shoulders, limbs, and cheeks. Keloids can invade surrounding healthy skin out of the original wound, usually causing pain, severe itch, malformation, and even dysfunction, seriously affecting the physical and mental health of patients (Bijlard et al., 2017). Therapeutic options for keloids are few and ineffective with common recurrence and deformities posttherapy (e.g., surgery, drug injection, and perioperative radiation therapy) (Qu et al., 2013; Gold et al., 2020; Lv et al., 2020). Therefore, there is an urgent need to explore and understand the aggressive progression of keloids to help identify novel effective treatment strategies and preventive methods.

Considering that keloid disease is of reticular dermal origin (Xue et al., 2022), KDFs serve as the predominant cell component in keloid tissue and have attracted much attention in keloid research. Dysregulated proliferation and apoptosis of fibroblasts are considered pivotal factors in the sustained hyperplasia of keloids (Shih et al., 2010), but the exact etiopathogenesis remains unclear. Studies have increasingly reported that epigenetics have important contributions to multifaceted processes of fibrosis, namely, DNA methylation, histone modification, and noncoding RNAs (miRNAs, lncRNAs, and circRNAs) (Yang et al., 2021). In particular, circRNAs, a new class of ncRNAs with covalently closed circular structures, have been shown to function in multiple biological processes with major roles as miRNA sponges, RNA-binding protein sponges, and polypeptide translation templates (Sinha et al., 2021; Zhao et al., 2021). For example, Zhang and the co-workers (Zhang et al., 2019) found that circHIPK3 is upregulated in a pulmonary fibrosis model and that si-circHIPK3 inhibits fibroblast proliferation by competitively binding to miR-338-3p. In colorectal cancer, circRHOBTB3 has been shown to exert suppressive effects on aggressiveness through the HuR/PTBP1 axis in both *in vivo* and *in vitro* experiments (Chen et al., 2021). Another study has identified that circ-SHPRH has an overlapping start-stop codon and is translated into a 146-aa polypeptide through IRES-mediated mechanisms in brain glioma (Zhang et al., 2018).

However, few studies have systematically evaluated circRNAs in keloid dermal fibroblasts (KDFs), and the function of dysregulated circRNAs remains to be further elucidated. Here, we identified differentially expressed (DE) circRNAs between KDFs and normal dermal fibroblasts (NDFs) by circRNA microarray analysis, which identified 327 DE circRNAs (195 upregulated and 132 downregulated) in KDFs. Furthermore, a circRNA-miRNA-mRNA network of verified candidates was established to understand the potential function of circRNAs in keloids. Finally, we found that hsa_circ_0006867 was significantly upregulated and promoted fibrosis in keloids. To our knowledge, this is the first report of the function and mechanism of hsa_circ_0006867 in keloids, and these data may offer a new understanding of the pathophysiologic mechanism in keloids.

## Results

### Identification of Dysregulated circRNAs in Keloids

**FIGURE 1:**
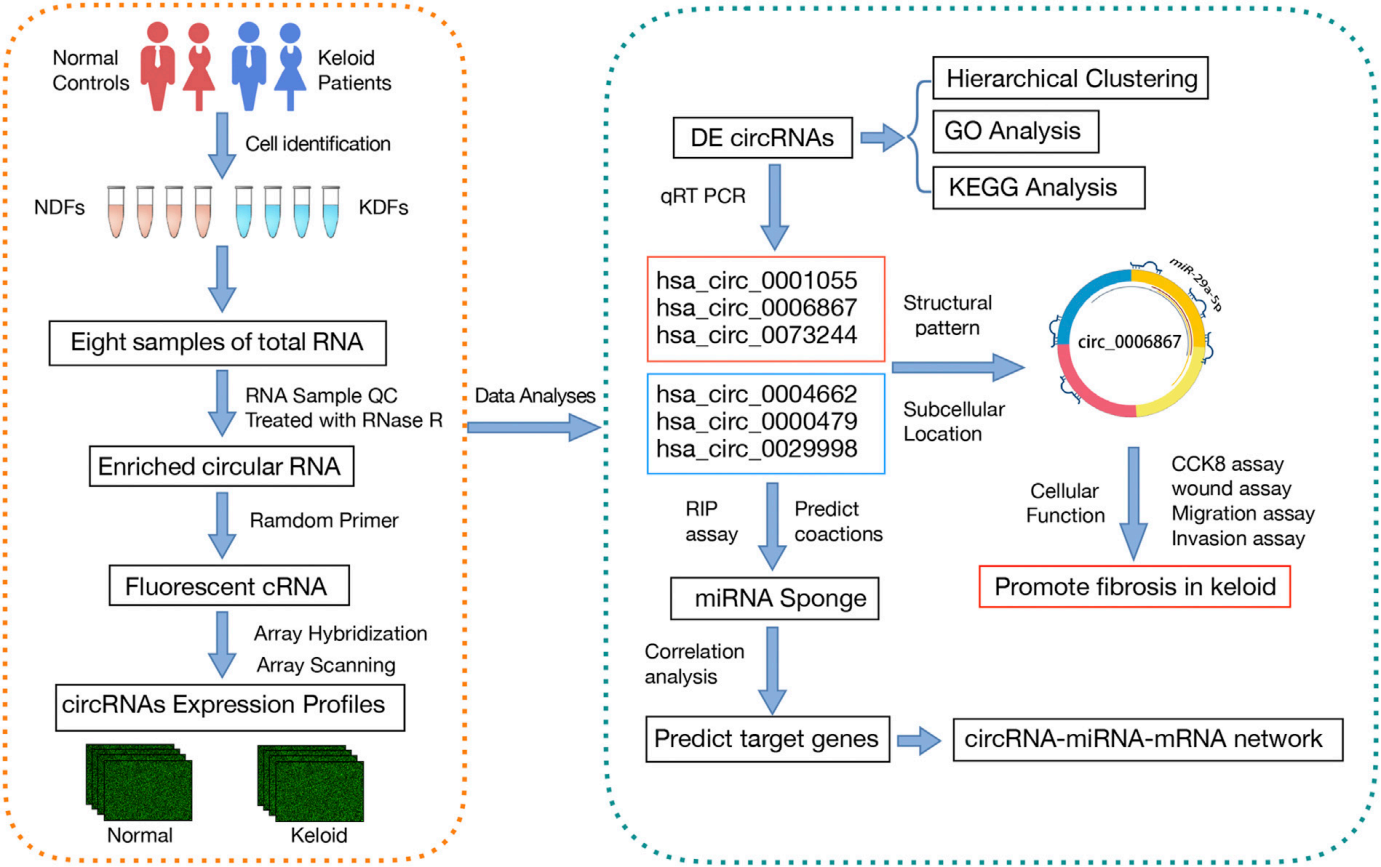
Detailed flowchart of the entire study. First, microarray analysis was performed to explore DE circRNAs in KDFs and NDFs. Then, GO and KEGG pathway analyses showed the potential functions of the DE circRNAs. six circRNAs were then verified by qRT–PCR, and a circRNA-miRNA-mRNA network was established for the validated circRNAs. Finally, we explored the cellular function and potential mechanism of hsa_circ_0006867.

**FIGURE 2:**
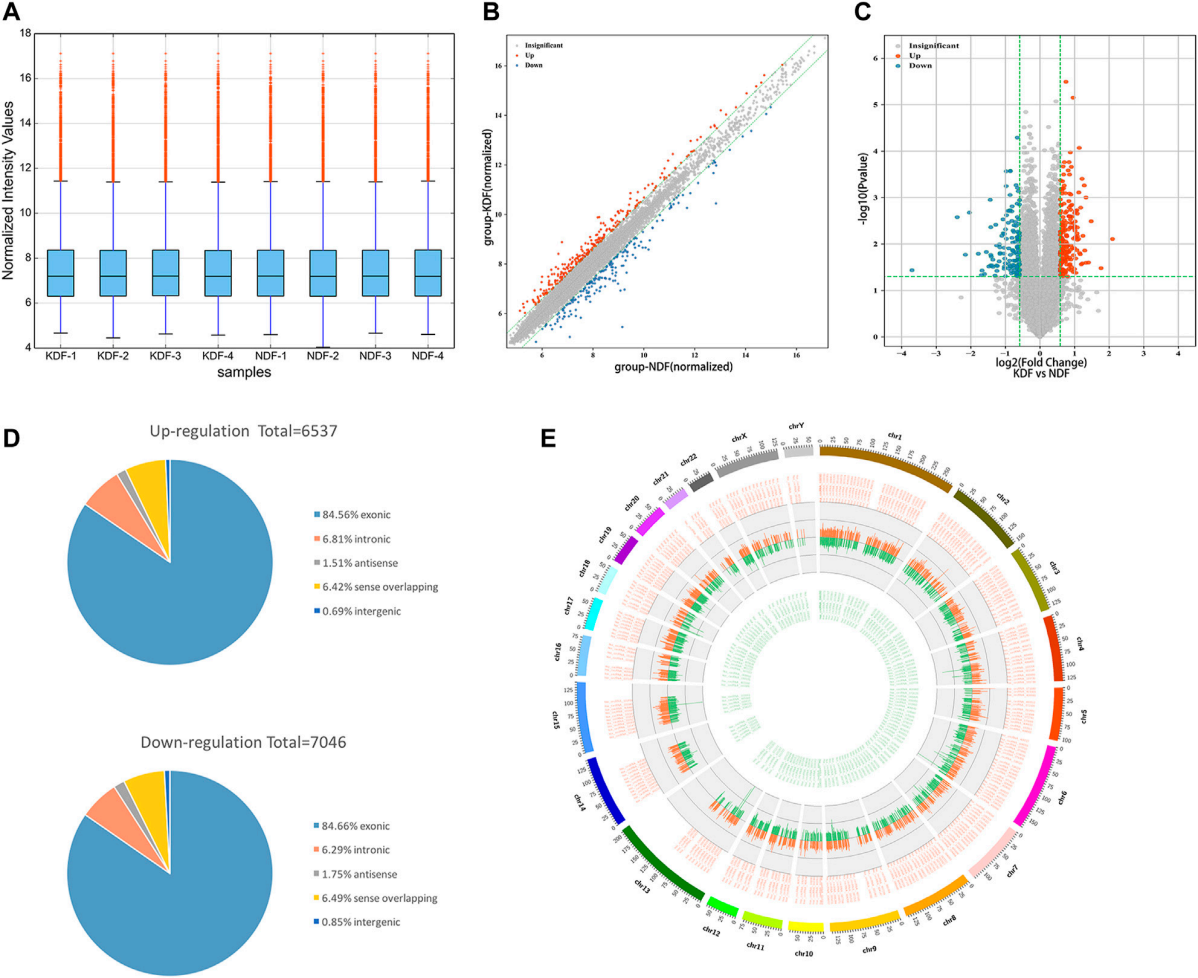
Expression profiles and characteristics of circRNAs in KDF and NDF samples as examined by microarray. **(A)** The box plot shows the distributions of the expression values after quantile normalization. **(B)** The scatter plot shows the difference in circRNA expression between KDFs and normal controls. The red dots above the green line and blue dots below the bottom indicate a more than 1.5-fold change (FC) in circRNAs between the two compared groups. **(C)** Volcano plot of differentially expressed circRNAs (fold change > 1.5 and *p* < 0.05). The red and blue dots represent upregulated and downregulated circRNAs, respectively. **(D)** The circRNA classification statistics show the percentage of each type among all differentially expressed circRNAs. **(E)** The Circos diagram depicts the location of all differentially expressed circRNAs on chromosomes. The orange/red and green lines represent upregulated and downregulated circRNAs, respectively. The segment length indicates the FC value.

The design of the present study is summarized in the flowchart shown in Figure 1. Our microarray profiling analyses detected a total of 13,583 circRNAs. After normalization of the microarray profile (Figure 2A), 327 circRNAs (fold change> 1.5, *p* < 0.05) showed differential expression between the two groups with 195 upregulated and 132 downregulated circRNAs. Scatter and volcano plots revealed the DE circRNAs (Figures 2B,C). The results revealed that more than 80% of circRNAs were derived from exons of the host gene (Figure 2D). A Circos diagram was applied to show the location of circRNAs on chromosomes (Figure 2E).

**FIGURE 3:**
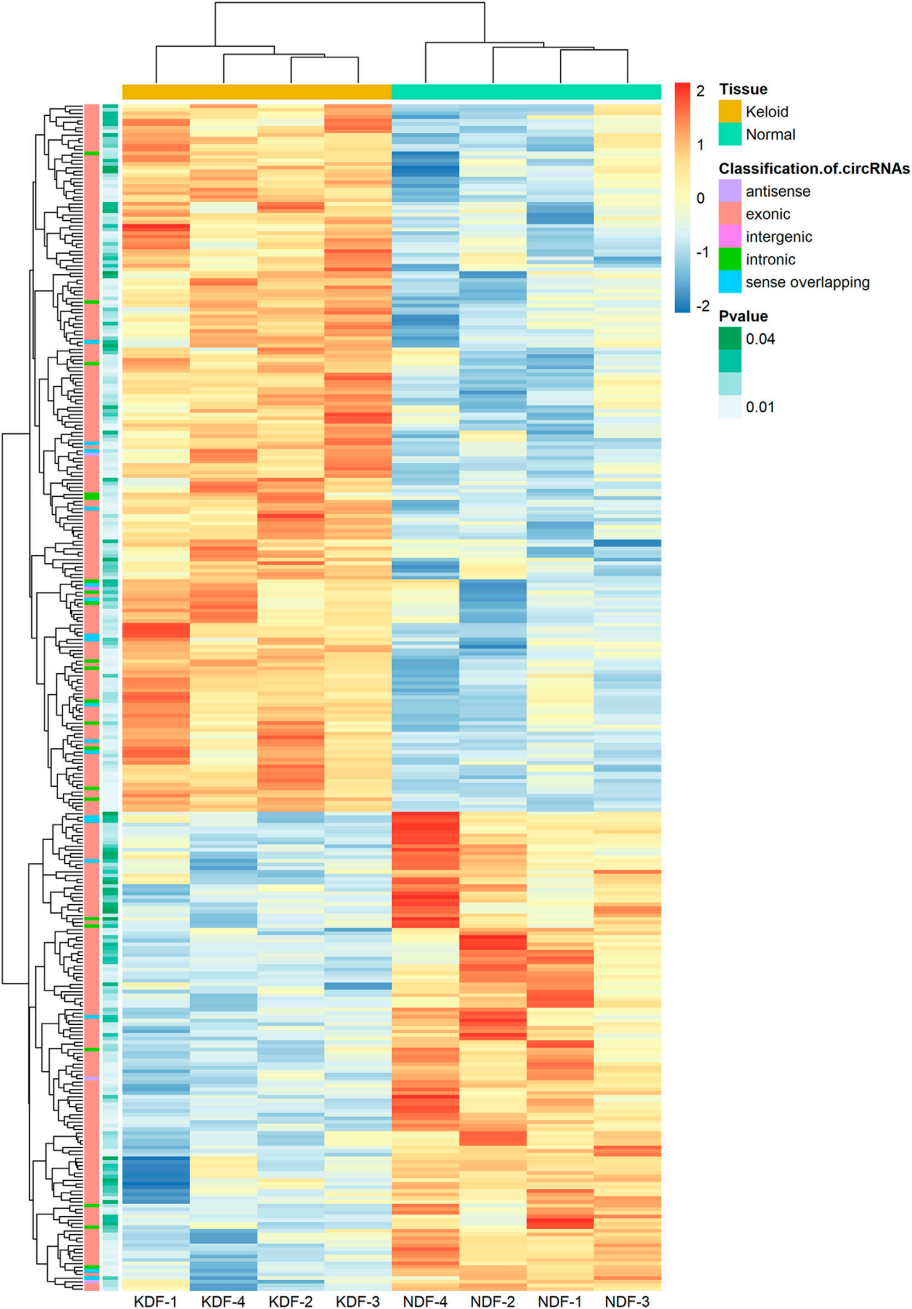
Heatmap of circRNA profiles from the microarray data showing distinguishable circRNA expression patterns. Red and blue indicate high and low expression, respectively, and the color scales represent expression values. The columns represent samples, and the rows represent circRNAs. The type of circRNA and *p* values of each analysis are shown in the first and second columns, respectively.

In addition, unsupervised hierarchical clustering analyses displayed the circRNA landscape to distinguish the KDF and NDF samples, which identified 327 dysregulated circRNAs (Figure 3). The top 20 significantly dysregulated circRNAs ranked by fold change between groups are listed in Table 1. The datasets presented in this study can be found in the Gene Expression Omnibus (GEO) site repositories (https://www.ncbi.nlm.nih.gov/geo/query/acc.cgi?acc=GSE184097).

**TABLE 1:** Top 20 significantly dysregulated circRNAs ranked by Fold Changes between KDF and NDF groups.

circRNA					
Name	circRNA_type	GeneSymbol	Fold change	Regulation	*p*-value
hsa_circRNA_406580	Exonic	LINC01060	4.2779339	Up	0.007793923
hsa_circRNA_102787	Exonic	AFF3	3.4009155	Up	0.033078187
hsa_circRNA_101118	Exonic	NTN4	2.7908248	Up	0.003235453
hsa_circRNA_103908	Exonic	EDIL3	2.6426199	Up	0.024996848
hsa_circRNA_103931	Exonic	FBN2	2.5145622	Up	0.025361528
hsa_circRNA_100478	Exonic	C1orf198	2.5143222	Up	0.000995353
hsa_circRNA_406083	Intronic	TASP1	2.454892	Up	0.013773615
hsa_circRNA_103752	Exonic	LRBA	2.4302802	Up	0.000547892
hsa_circRNA_073803	Exonic	FBN2	2.3661298	Up	0.006674997
hsa_circRNA_100686	Exonic	ATRNL1	2.3380092	Up	0.026210801
hsa_circRNA_029998	Exonic	CCDC169	12.9885222	Down	0.03661059
hsa_circRNA_004662	Exonic	SOD2	5.2485949	Down	0.002644992
hsa_circRNA_000479	Exonic	EPSTI1	4.4556299	Down	0.016988318
hsa_circRNA_406490	Exonic	PRKG2	4.1092578	Down	0.002104969
hsa_circRNA_004658	Exonic	EMILIN2	3.4392155	Down	0.016198436
hsa_circRNA_405665	Exonic	GREB1L	3.22882	Down	0.046681385
hsa_circRNA_077755	Exonic	GJA1	3.1899219	Down	0.037231433
hsa_circRNA_088194	Exonic	TNC	3.0414237	Down	0.044245795
hsa_circRNA_405647	Exonic	COLEC12	2.9281235	Down	0.014837202
hsa_circRNA_070520	Exonic	SLC39A8	2.7837685	Down	0.030511757

### Functions of Differentially Expressed circRNAs

**FIGURE 4:**
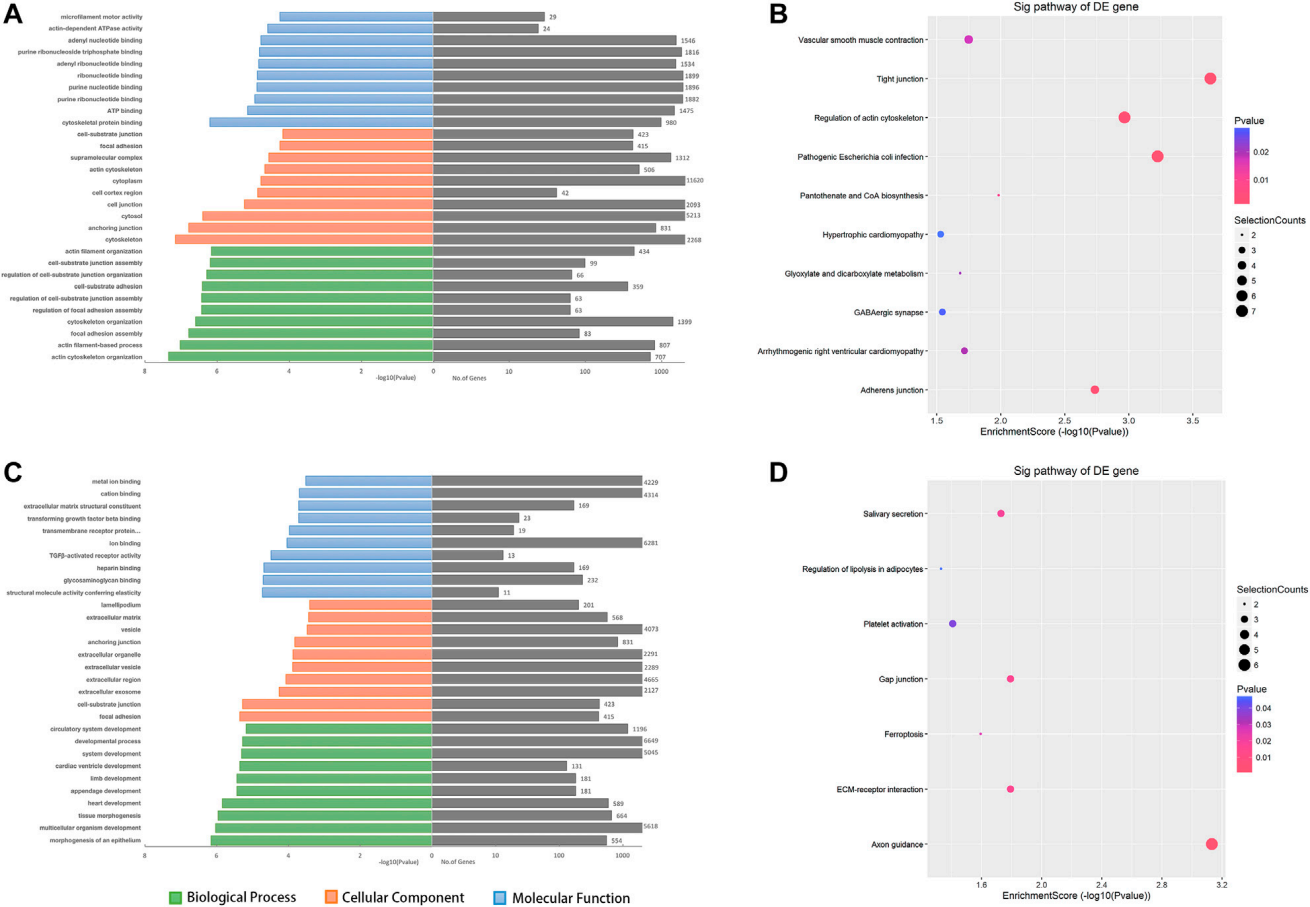
The top 10 GO and KEGG pathway enrichment terms for the predicted target genes of DE circRNAs. GO analysis covered three domains, namely biological processes, cellular components, and molecular functions. The KEGG plot shows the top enrichment score value of the significantly enriched pathway. **(A, B)** The upregulated - and **(C, D)** downregulated circRNAs were analyzed. Abbreviations: ORF, open reading frame.

Because genes with similar functions share the same expression patterns, we focused on the alterations of coding genes between groups to reveal the behavior of the aberrantly expressed circRNAs. To further explore the functions of the dysregulated circRNAs, the host genes of the DE circRNAs were selected to perform Gene Ontology (GO) enrichment analyses. GO analyses of the target genes covered three domains as follows: biological process (BP), cellular component (CC), and molecular function (MF). Regarding the upregulated circRNAs, actin cytoskeleton organization, cytoskeleton, and cytoskeletal protein binding were the most abundant terms in BP, CC, and MF, respectively (Figure 4A). Kyoto Encyclopedia of Gene and Genomes (KEGG) pathway analysis indicated that tight junctions and regulation of the actin cytoskeleton were the top two related functions of the upregulated circRNAs (Figure 4B). Moreover, GO analyses indicated that the downregulated circRNAs were mainly related to morphogenesis of the epithelium, focal adhesion, and structural molecule activity conferring elasticity, and KEGG analyses indicated that axon guidance and ECM−receptor interaction were the top two related functions of downregulated circRNAs (Figures 4C,D). Maps of the most affected pathways are shown in supplementary Supplementary Figures S1, S2.

### Verification of the Differentially Expressed circRNAs

**FIGURE 5:**
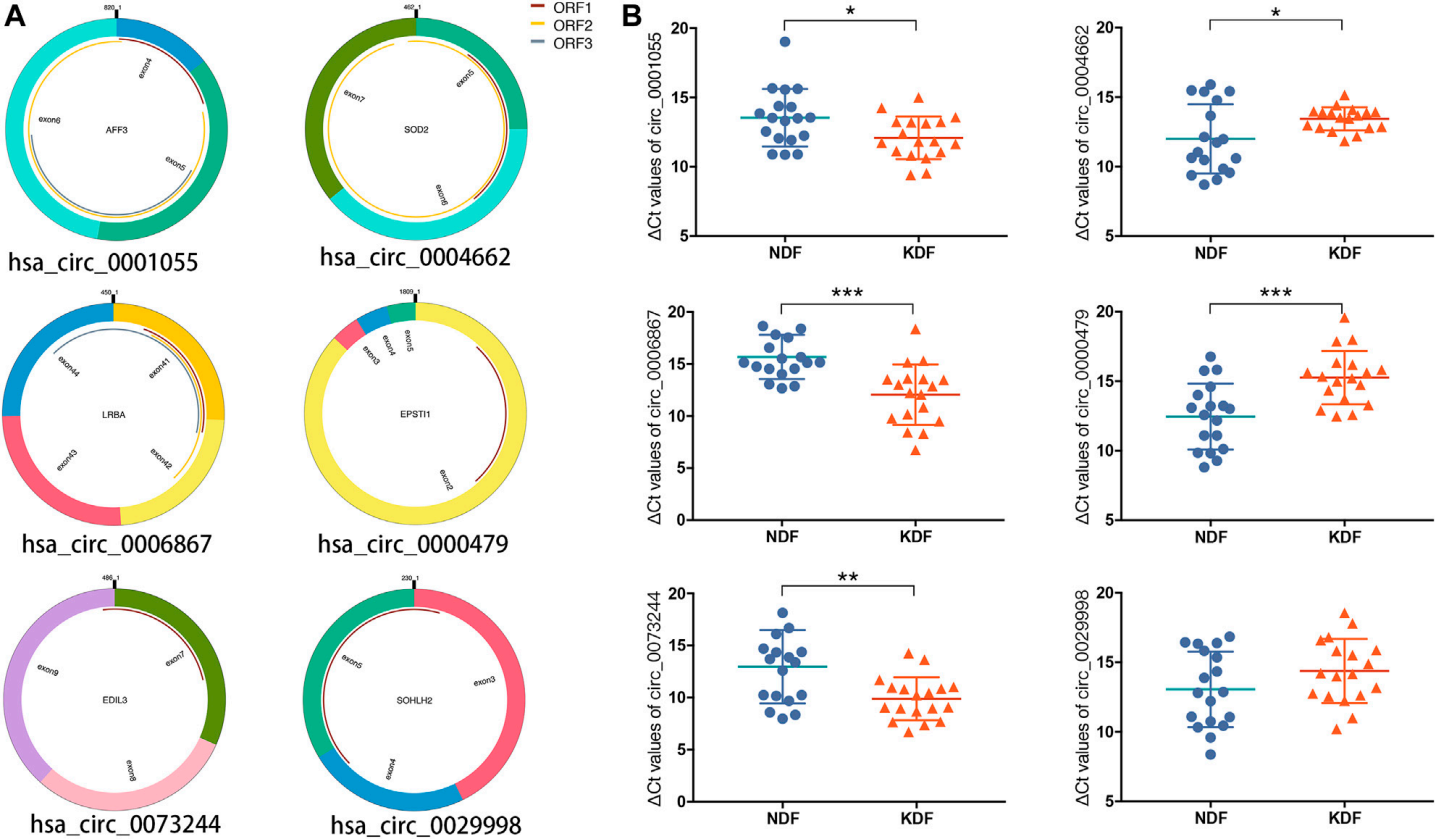
Characteristics and verification of six candidate circRNAs. **(A)** Structural patterns and **(B)** qRT–PCR results of upregulated circRNAs (left), including hsa_circ_0001055, hsa_circ_0006867, and hsa_circ_0073244, as well as three downregulated circRNAs, including hsa_circ_0004662, hsa_circ_0000479, and hsa_circ_0029998.

After considering the fold change, *p* value, and raw data, we selected three significantly upregulated circRNAs (hsa_circ_0001055, hsa_circ_0006867, and hsa_circ_0073244) and three significantly downregulated circRNAs (hsa_circ_0004662, hsa_circ_0000479, and hsa_circ_0029998) for qRT–PCR validation. As shown in Figure 5A, the structure of the six circRNAs was visualized based on circPrimer 2.0, and their relative intensity values were evaluated by microarray analysis. The PCR primers are shown in Table 2. By expanding the sample numbers, the qRT–PCR results showed that the changes in the six circRNAs were consistent with the microarray data, but the expression of hsa_circ_0029998 between keloids and normal skin did not differ statistically (Figure 5B). Therefore, five verified DE circRNAs were selected for further analysis by bioinformatics methods.

**TABLE 2:** Primer sequences used for qRT-PCR analysis of circRNA and mRNA levels.

Name	Primer F (5′-3′)	Primer R (5′-3′)
hsa_circ_0001055	GAGTCCCTGCCAGCAAGC	ATC​TGG​TTC​ATA​GAC​ACT​CC
hsa_circ_0006867	AAC​TTC​AGA​GAT​TTG​TCC​AAG​A	TCT​CCA​GGG​CTG​TAT​TTT​GC
hsa_circ_0073244	TCA​CAA​TGG​TTA​CAG​ATA​AAT​TTG	TGT​AGG​CAA​TTT​TGT​AGG​ATT​TT
hsa_circ_0004662	CCA​CGA​TCG​TTA​TGC​TGA​GA	TAG​GGC​TGA​GGT​TTG​TCC​AG
hsa_circ_0000479	AAA​ATG​AAG​GCA​ATT​CAG​AGA​GA	AAA​ATG​AAG​GCA​ATT​CAG​AGA​GA
hsa_circ_0029998	GAG​AAT​CTA​GTG​AAA​ACG​AAT​CCT​TA	AAG​CCT​TTG​ATT​CTT​GTT​CCA
hsa_miR_29a-5p	GCT​GCG​ACT​GAT​TTC​TTT​TGG​T	AGT​GCA​GGG​TCC​GAG​GTA​TT
GAPDH	GAACGGGAAGCTCACTGG	GCC​TGC​TTC​ACC​ACC​TTC​T
U6	GGA​ACG​ATA​CAG​AGA​AGA​TTA​GC	TGG​AAC​GCT​TCA​CGA​ATT​TGC​G

### Establishment of the circRNA-miRNA-mRNA Network

**FIGURE 6:**
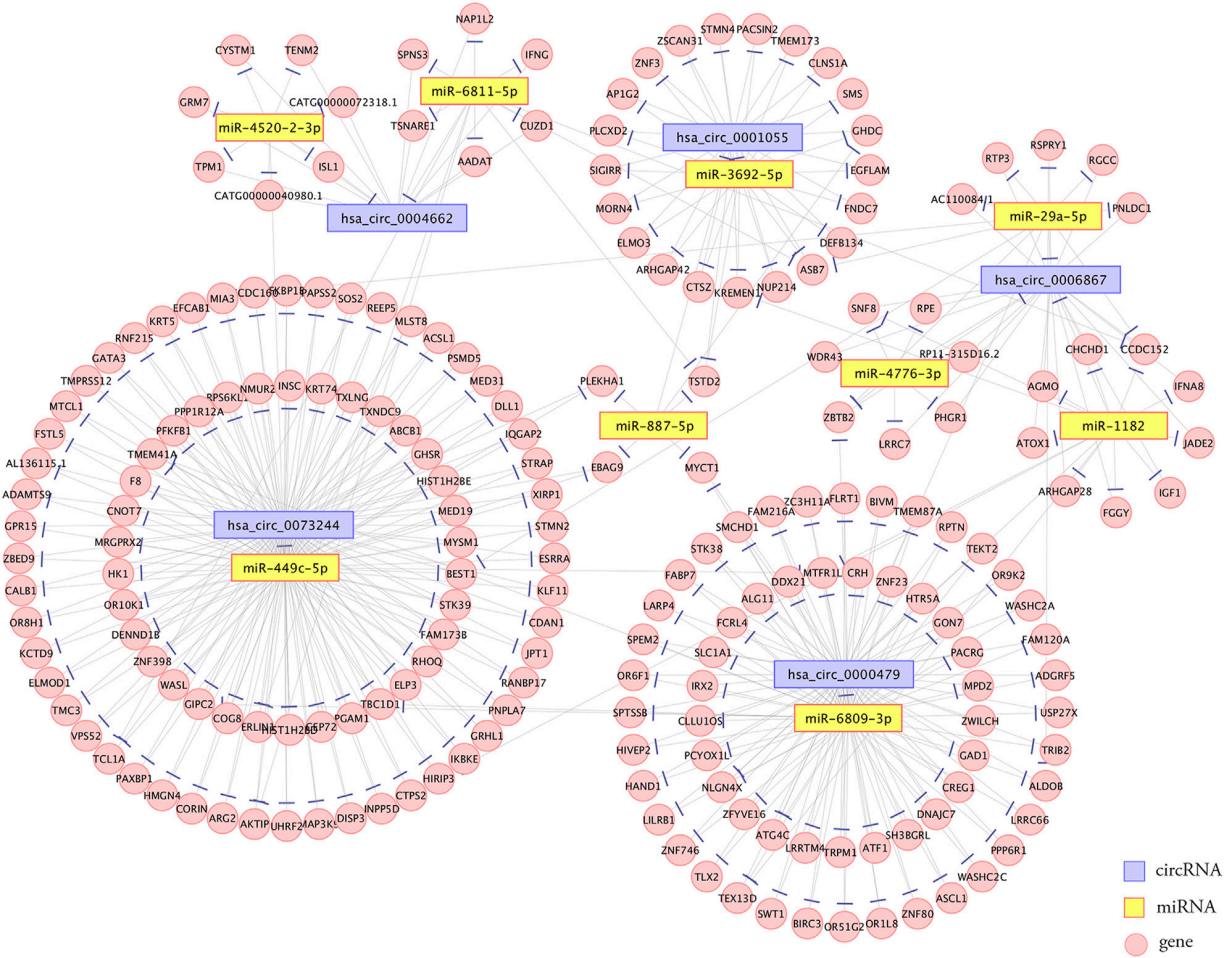
Establishment of the circRNA-miRNA-mRNA interaction network. The network contained five dysregulated circRNAs, nine miRNAs, and 235 correlated mRNAs. Edges with T-shaped arrows represent directed relationships. Nodes with purple, yellow, and pink color represent circRNAs, miRNAs, and mRNAs, respectively.

The competing endogenous RNA (ceRNA) hypothesis states that RNA transcripts cross-regulate each other by competing for shared miRNAs (Qi et al., 2015). Therefore, we applied Arraystar’s prediction software to construct ceRNA networks of the five verified DE circRNAs, which linked the function of protein-coding mRNAs with that of noncoding RNAs, including miRNAs and circRNAs. A total of 235 significantly negatively correlated mRNAs were regarded as the potential target genes of the nine miRNAs. The predicted circRNA-miRNA-mRNA network is shown in Figure 6. The detailed information of the circRNAs-miRNAs-mRNAs interaction network is provided in Supplementary Table S1.

### Inhibition of hsa_circ_0006867 Suppresses Keloid Cell Proliferation, Migration, and Invasion *in Vitro*

**FIGURE 7:**
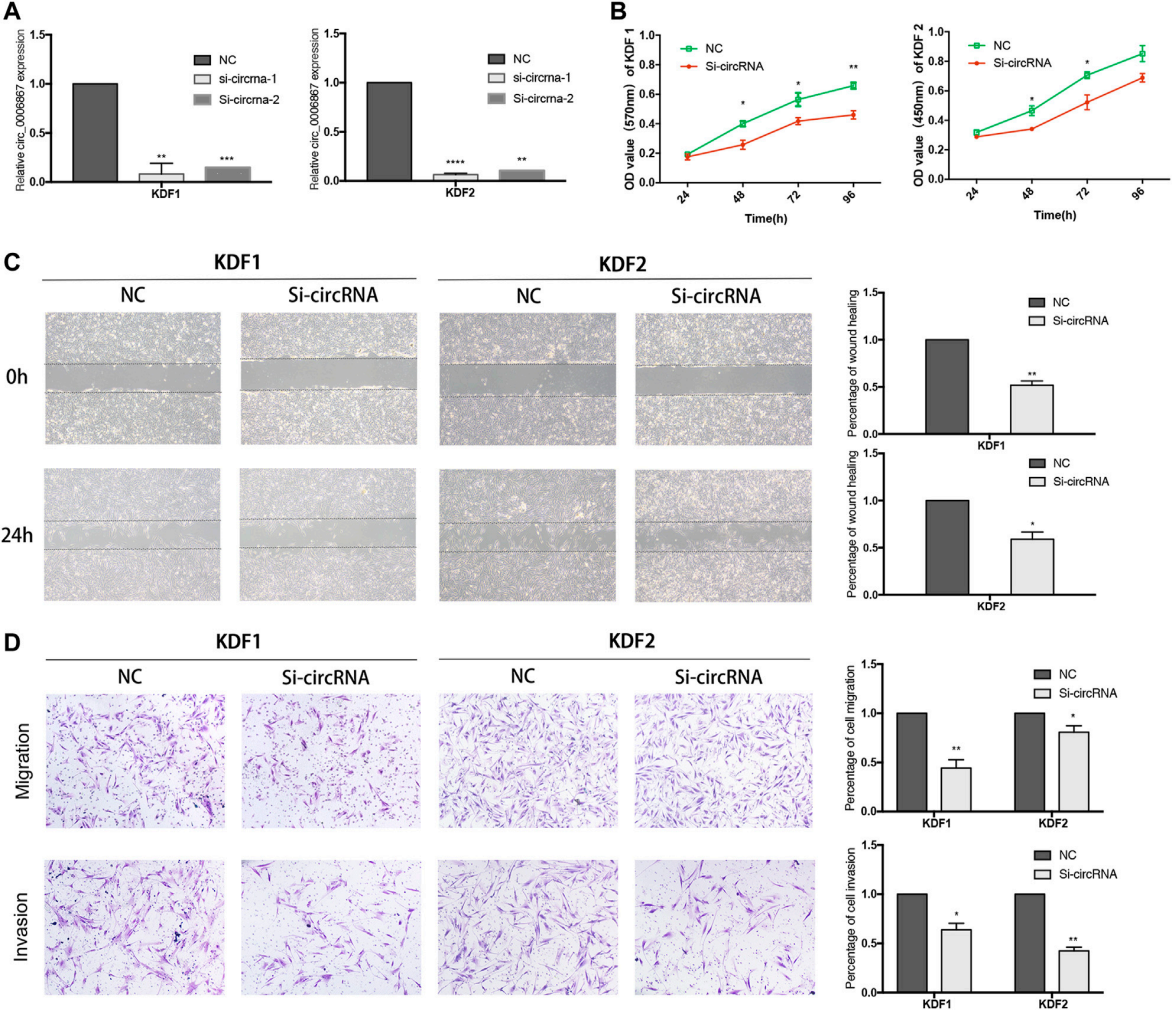
Inhibition of hsa_circ_0006867 suppresses keloid cell proliferation, migration, and invasion. **(A)** Effect of siRNAs targeting hsa_circ_0006867. **(B)** CCK-8 assays. **(C)** Scratch wound-healing assays. **(D)** Transwell migration and invasion assays. *p < 0.05, **p < 0.01, and ***p < 0.001.

Because hsa_circ_0006867 was increased significantly in keloids (Figure 5B), we investigated the biological functions of hsa_circ_0006867. Two siRNAs targeting the back-splice junction sites of hsa_circ_0006867 were designed and verified significantly knockdown the expression of hsa_circ_0006867 (Figure 7A). The CCK-8 assay showed that inhibition of hsa_circ_0006867 significantly suppressed cell proliferation in both KDF1 and KDF2 cells (Figure 7B). Transwell assays and wound-healing assays indicated that silencing hsa_circ_0006867 significantly suppressed the invasion and migration ability of both KDF1 and KDF2 cells (Figures 7C,D). These results indicated that hsa_circ_0006867 plays a key role in the suppression of proliferation, invasion, and migration of KDF cells *in vitro*.

### Hsa_circ_0006867 Functions as a Sponge for miRNAs

**FIGURE 8:**
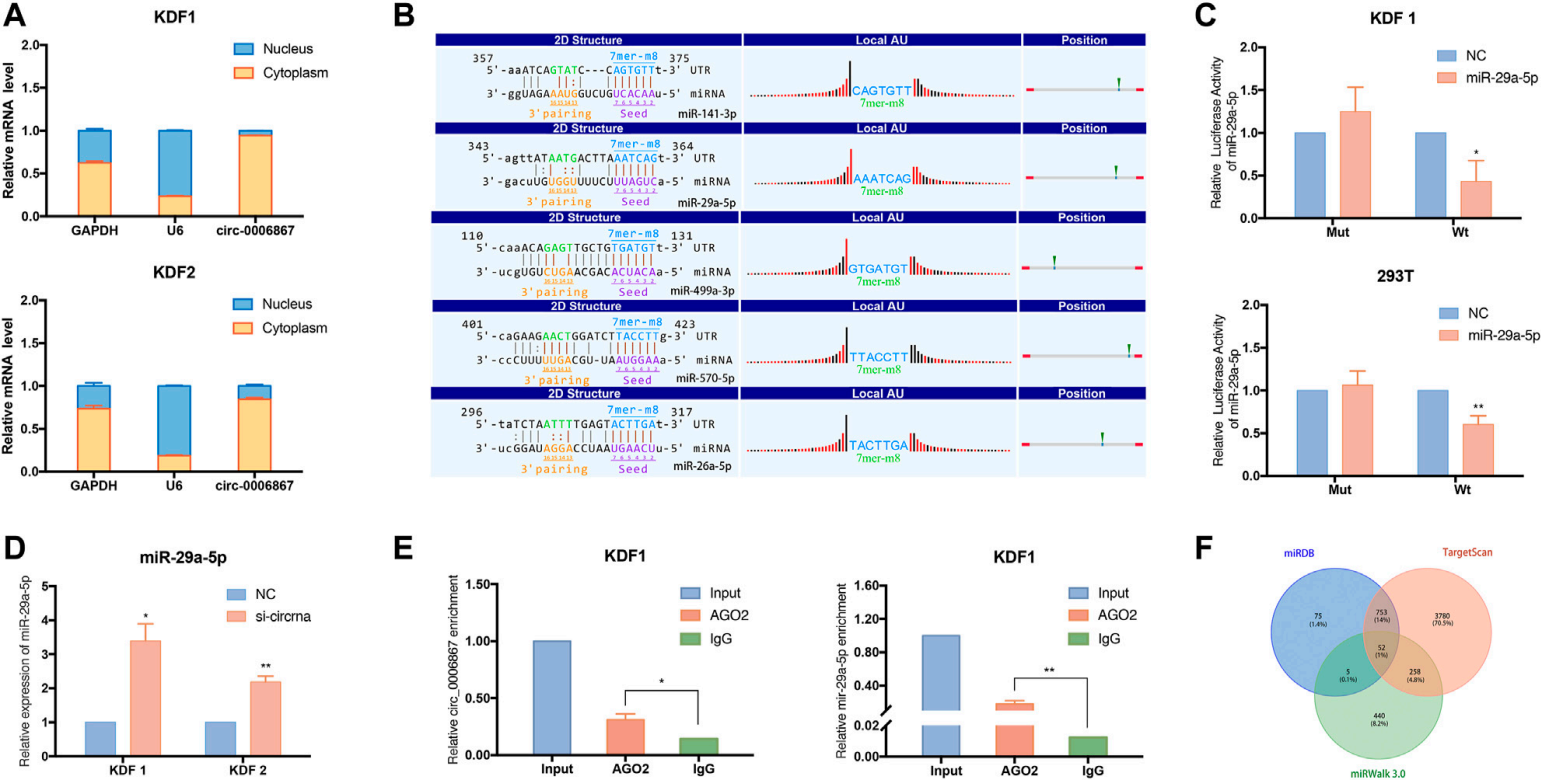
Exploration of the potential regulatory mechanism of hsa_circ_0006867 in keloids. **(A)** Nuclear and cytoplasmic fractionation assays verified the localization of hsa_circ_0006867 in KDF1 and KDF2 cells. **(B)** The five highest ranking target miRNAs of hsa_circ_0006867 and annotation of their circRNA/miRNA interactions. **(C)** The dual-luciferase reporter assay was performed using 293T and KDF1 cells after cotransfection. **(D)** The expression of miR-29a-5p was significantly upregulated after silencing hsa_circ_0006867. **(E)** The RIP assay showed that hsa_circ_0006867 and miR-29a-5p were enriched in the presence of AGO2 antibody. **(F)** The Venn plot showed the overlap of the predicted mRNA target of miR-29a-5p based on the datasets from miRDB, TargetScan, and miRWalk 3.0.

Because circRNAs are mainly located in the cytoplasm and may act as sponges for miRNAs, we explored the subcellular localization of hsa_circ_0006867 in KDF cells. We extracted total RNA from the nucleus and cytoplasm, and we then applied qRT–PCR to measure the expression of hsa_circ_0006867. The results indicated that hsa_circ_0006867 was mainly located in the cytoplasm in KDF1 and KDF2 cells (Figure 8A). To determine the potential mechanism of hsa_circ_0006867 and the common target miRNAs, we analyzed the five highest ranking target miRNAs of hsa_circ_0006867 and annotated their circRNA/miRNA interactions (Figure 8B). Subsequently, the wild-type or mutated seed sequence of hsa_circ_0006867 was designed to determine whether there is a direct interaction. A dual-luciferase reporter assay in both 293T cell lines and KDF1 cells indicated that the miR-29a-5p mimic inhibited the luciferase activity of wild-type vectors (Figure 8C). Moreover, the expression of miR-29a-5p was upregulated after silencing hsa_circ_0006867 (Figure 8D).

Considering that miRNAs silence the expression of target genes by assembling with Argonaute (AGO) 2, we performed a RNA-Binding Protein Immunoprecipitation (RIP) assay in KDF cells using AGO2 antibodies. Compared to the IgG group, hsa_circ_0006867 was significantly enriched in the presence of AGO2 antibody, suggesting that hsa_circ_0006867 may serve as a platform for miRNA binding to AGO2 (Figure 8E). Thus, the above results demonstrated the potential of hsa_circ_0006867 to sponge miRNAs.

We also found that miR-29a-5p was enriched in the AGO2 group in the RIP assay (Figure 8E). The overlapping genes of the related mRNAs of miR-29a-5p from miRDB, TargetScan, and miRWalk 3.0 were analyzed and subsequently visualized using a Venn diagram (Figure 8F). For miR-29a-5p, 52 common genes were identified. Of these, *KCCN3* may be a promising potential target. Overall, these findings suggested that hsa_circ_0006867 may play a regulatory function in keloid progression as a sponge for miR-29a-5p.

## Discussion

As benign skin fibroproliferative tumors, keloids are characterized by a typical fibrosis state and some cancer-like features (Gold et al., 2020). A previous study has reported that circRNAs participate in the progression of fibrosis in various organs, including the skin, heart, liver, lung, and kidney (Dong et al., 2021; Wan et al., 2021). For instance, hsa_circ_0045272 may negatively regulate apoptosis and interleukin-2 secretion in T cells of systemic lupus erythematosus patients [21]. However, the role of circRNAs in the pathogenesis of keloids remains unknown. In the present study, we collected keloid tissues from different anatomical locations and cultured them as KDFs. By utilizing circRNA microarray analysis, we compared the circRNA profiles between KDFs and NDFs, and we identified 327 DE circRNAs (195 upregulated and 132 downregulated). The functions of the DE circRNAs were predicted by GO and KEGG analyses. Based on qRT–PCR results, five verified DE circRNAs were selected to construct the circRNA-miRNA-mRNA network. Moreover, we found that hsa_circ_0006867 may promote the aggressive progression of keloids by acting as a miRNA sponge.

GO and KEGG pathway enrichment analyses were performed to evaluate the functions of target genes at the molecular level. According to GO analysis for upregulated circRNAs, actin cytoskeleton organization, cytoskeleton, and cytoskeletal protein binding were the most enriched terms of biological process, cellular component, and molecular function, respectively. A previous study has shown that pathological scarring in the form of keloids and hypertrophic scars is associated with the dysfunction of cellular mechanoreceptors in the skin (such as the cytoskeleton and cell adhesion molecules) (Ogawa and Hsu, 2013). Organization of the cytoskeleton can perceive mechanical forces (Wong et al., 2011) and influence the stiffness and rigidity of the ECM as well as moderate cell motility and proliferation (Wang et al., 2015). In addition, the KEGG results also showed the important role of the cytoskeleton. Regarding downregulated circRNAs, morphogenesis of the epithelium showed the highest relevance in GO analyses. It is well known that epithelial cells transform into myofibroblasts and fibroblasts via the epithelial-mesenchymal transition (EMT) process (Yuan et al., 2019; Huang and Ogawa, 2020). Chen and the co-workers (Chen et al., 2020) found that circ_0008450 promotes proliferation and processing in human keratinized epithelial cells through the TGF-β/Smad/Runx3 axis. Moreover, the ECM-receptor interaction pathway has been suggested to be associated with downregulated circRNAs in KEGG pathway maps. Together, these findings may provide information for the research direction of DE circRNAs in keloids.

In the present study, six circRNAs (3 up- and three downregulated) were validated by qRT–PCR in 18 sample groups according to the following standards: circRNA mature sequence between 200 and 1,000 bp with top fold changes and binding sites with miRNAs. The results demonstrated that five circRNAs (hsa_circ_0001055, hsa_circ_0006867, hsa_circ_0073244, hsa_circ_0004662, and hsa_circ_0000479) showed significant changes, and the expression of hsa_circ_0029998 was altered but the changes were not statistically significant. Consequently, a circRNA-miRNA-mRNA network, including five circRNAs, nine miRNAs, and 235 correlated mRNAs, was established.

We then performed cellular function experiments to further explore the potential mechanism of candidate circRNAs in keloid formation. The results suggested that the inhibition of hsa_circ_0006867 significantly influenced the proliferation, migration, and invasion of KDFs *in vitro*, indicating that it may be a potential biomarker for keloids. Hsa_circ_0006867 is encoded from the 41st to 45th exons of *LRBA* containing 450 bp, and it was found to be more stable than the baseline gene. The present study verified that hsa_circ_0006867 was mainly located in the cytoplasm of KDFs where it sponged miRNAs. Previous studies have shown that circRNAs regulate miRNAs by interacting with miRNA response elements, which can competitively suppress miRNA activity, thereby regulating the expression of downstream genes (Salmena et al., 2011). We next predicted the circRNA/miRNA interaction, which indicated that miR-29a-5p, miR-141-3p, miR-499a-3p, miR-570-5p, and miR-26a-5p were involved (Figure 8C). A dual-luciferase reporter assay indicated that miR-29a-5p directly interacted with hsa_circ_0006867, and silencing hsa_circ_0006867 also significantly induced upregulation of miR-29a-5p.

An anti-AGO2 RIP assay was also performed to identify the relationship between hsa_circ_0006867 and these miRNAs. It is well known that the AGO family is composed of four enzymes, namely, AGO1, AGO2, AGO3, and AGO4, which are ubiquitously expressed in mammals and are all capable of loading small RNA (Ross and Kassir, 2014). Among them, AGO2 is called a “catalytic engine” that drives mRNA cleavage at miRNA target sites (Tam et al., 2008). The expression of hsa_circ_0006867 and miR-29a-5p was enriched with AGO2, indicating that hsa_circ_0006867 may bind to the AGO2 protein and thus regulate the potential target, miR-29a-5p. Next, we applied Venn analysis to identify the downstream gene of miR-29a-5p based on three databases (miRDB, TargetScan, and miRWalk 3.0). A previous study has shown that miR-29a-5p is downregulated in endometrial cancer and inhibits cancer-derived cell proliferation (Jiang et al., 2018). However, the role of miR-29a-5p in keloid progression has not yet been reported. In addition, the predicted target gene, *KCCN3,* has been found to be involved in matrix degradation, and blockade of *KCCN3* inhibits migration, induces cells depolarization, and reduces intracellular Ca2+ in a highly metastatic mammary cancer cell line (Potier et al., 2006; Shenton et al., 2014). Thus, KCCN3 may be a promising potential target of miR-29a-5p, but further experiments are needed.

In summary, the present findings suggested that hsa_circ_0006867 may promote the progression of keloids via the miR-29a-5p/KCCN3 axis. However, the potential regulatory mechanism needs to be further verified.

## Materials and Methods

### Patient Samples

Keloid and normal control tissues were collected from 36 patients who had undergone surgery at the Department of Plastic Surgery of First Affiliated Hospital, School of Medicine, Zhejiang University from 2017 to 2020. All patients were diagnosed by pathological examination and had not been treated previously. Eight samples were enrolled in microarray profiling analyses. The clinical and demographic information of the patients is displayed in Table 3. Every patient signed an informed consent form before surgery, and all experiments were performed after approval by the Clinical Research Ethics Committee of the First Affiliated Hospital, School of Medicine, Zhejiang University (No. 2014058; February 27, 2014).

**TABLE 3:** Clinical information of included subjects.

Characteristics	Keloid					Normal skin
Total number	18					18
Gender (Male/Female)	7/11					6/12
Age(y),mean ± SD	25.78 ± 4.56					28.39 ± 8.75
**Sample location**	**Location**	**No.of patients**	**Percent of patients (%)**	**Location**	**No.of patients**	**Percent of patients (%)**
	Auricle	8	44.4%	Face	5	27.8%
	earlobe	6	33.3%	Chest	5	27.8%
	Face	1	5.6%	foreskin	3	16.7%
	Chest	1	5.6%	Lower limb	3	16.7%
	upper limb	1	5.6%	Upper limb	1	5.6%
	Back	1	5.6%	Back	1	5.6%
cirRNA micro-array	4			4		
Gender (Male/Female)	2/2			2/2		
Age(y),mean ± SD	30.25 ± 4.56			31.00 ± 5.03		
**sample location**	**Location**	**No.of patients**	**Percent of patients (%)**	**Location**	**No.of patients**	**Percent of patients (%)**
	earlobe	1	25.0%	Face	1	25.0%
	Auricle	1	25.0%	Lower limb	1	25.0%
	Uper limb	1	25.0%	Upper limb	1	25.0%
	Chest	1	25.0%	Chest	1	25.0%

### Cell Culture and Transfection

Primary human dermal fibroblasts were cultured according to a previously established protocol (Pang et al., 2019). Tissue samples derived from donors were washed three times using PBS and minced into pieces after separating the epidermis. The tissue pieces were digested with 2.0 mg/ml type I collagenase (Solarbio, China) and trypsin (Gibco, United States). Cells were cultured in DMEM (Servicebio, China), containing 10% FBS (Gibco, United States), penicillin (100 U/ml), and streptomycin (100 μg/ml), in 5% CO_2_ at 37°C in an incubator. Fibroblasts were used from passage three to nine.

The siRNAs against hsa_circ_0006867 and the negative control were synthesized by RiboBio Co., Ltd. (China) and validated by qRT–PCR. The primer sequences were as follows: si-hsa_circ_0006867-1 (sense), 5′- GAGAUUUGUCCAAGAUCUUdtdt-3′; and si-hsa_circ_0006867-2 (sense), 5′-UCAGAGAUUUGUCCAAGAUdtdt-3′. To achieve better knockdown efficiency and fewer off-target effects, two siRNAs were merged together as a siRNA pool to cotransfect KDFs in the interference experiments. Following the manufacturer’s instructions, 50 nM siRNA was transfected into HKFs using jetPRIME transfection reagent (Polyplus, Illkirch, France).

### Circular RNA Microarray Detection and Differential Expression Analysis

Detailed information for the KDFs and NDFs used for microarray detection is listed in Table 3. Total RNA was extracted from cells using TRIzol reagent (Invitrogen, United States) and quantified using a NanoDrop ND-1000 (Thermo Fisher, United States). Total RNA was treated with ribonuclease R (Epicenter, United States) to remove linear RNAs and enrich circular RNAs (circRNAs). The enriched circRNAs were then amplified and transcribed into fluorescent cRNA utilizing a random priming method (Super RNA Labeling Kit, Arraystar, Rockville, United States). The labeled cRNAs were hybridized onto the Arraystar Human circRNA Array V2 (8 × 15K, Arraystar). After cleaning the slides, the arrays were scanned by the Agilent Scanner G2505C.

Agilent feature extraction software (version 11.0.1.1) was applied to analyze the acquired array images. We then used the limma package in R software for quantile normalization and microarray analysis. CircRNAs with a *p* value <0.05 and fold change ≥1.5 were considered to be significantly differentially expressed and were then identified through volcano plot filtration. The expression patterns of distinguished circRNAs were demonstrated using hierarchical clustering.

### GO and KEGG Analyses

GO analysis (http://www.geneontology.org) was conducted to further explore the potential biological functions of the host genes of the identified circRNAs. Kyoto Encyclopedia of Gene and Genomes (KEGG) pathway analysis (http://www.genome.jp/kegg/) revealed the signaling pathways based on the identified target genes of circRNAs.

### Quantitative Real-Time Polymerase Chain Reaction

Total RNA was extracted from cells and tissue samples with TRIzol reagent (Invitrogen, United States), and 500 ng of RNA was amplified and transcribed into cDNA using a PrimeScript RT reagent Kit (Takara, China). Specific primers covering circRNA back-splice junctions (sequences are shown in Table 2) were designed and synthesized by RiboBio Co., Ltd. (Guangzhou, China). Following the manufacturers’ instructions, circRNA and mRNA expression was measured by qRT–PCR using the SYBR Green Kit (Takara, China). The expression of each circRNA was analyzed using the 2-ΔΔCt method and normalized to the *GAPDH* gene.

### Prediction of the circRNA-miRNA-mRNA Pathway

We constructed the circRNA-miRNA-mRNA network based on the ceRNA hypothesis to predict the potential target miRNAs of the selected circRNAs. The downstream genes of miRNAs were predicted by two prediction algorithms, namely, TargetScan (http://www.targetscan.org/) and MiRanda (http://www.microrna.org/). Thereafter, cross-analysis of the two datasets was performed to identify the possible competing miRNAs using Arraystar’s commercial miRNA coaction prediction software. Finally, ceRNA networks were established using 5 DE circRNAs and predicted miRNAs and mRNAs.

### Cell Proliferation Assay

The CCK-8 assay determined the cell proliferation capacity at different times according to the manufacturer’s instructions. At 24, 48, 72, and 96 h, 200 µL of CCK-8 solution was added to each well followed by incubation at 37°C for 2 h. The OD values at 450 nm were measured using a microtiter plate reader. Each group was set up with three replicates.

### Transwell Experiment

The migration and invasion abilities of different groups were assessed by the Transwell assay. Transfected cells in 300 µL of serum-free DMEM were seeded in the bottom of the Transwell chamber (8 μm pore size; BD Falcon, Corning NY, United States) with or without Matrigel (Corning) for invasion or migration assays. The lower chamber contained 800 µL of serum-containing medium. After 48 h of incubation, cells in the upper chamber were removed, and the membrane was fixed with fixation solution for 4 min and stained with staining solution for 6 min (Wright-Giemsa Stain Kit, Nanjing Jiancheng Bioengineering Institute, China). The stained cells were imaged and counted in five fields of view using a phase contract microscope (Olympus, Japan).

### Scratch Wound-Healing Assay

We used a culture insert (Ibidi, Germany) for cell scratch wound-healing experiments. First, transfected cells were seeded into the cell insert at a suitable density and cultured overnight. The cell insert was then removed and washed twice with PBS. Cells were imaged 0 and 24 h, and the width of the wound was measured. The wound width in each group was measured in three replicates.

### Nuclear and Cytoplasmic Fractionation

Cytoplasmic and nuclear RNAs were separated using a PARIS™ kit (Invitrogen, United States). Following the manufacturer’s instructions, 1 × 10^7^ fresh cells were washed in PBS, fully resuspended in ice-cold cell fractionation buffer, and incubated on ice. The supernatant (cytoplasm) and pellet (nucleus) were separated by centrifugation. The nuclear fractions were then lysed by cell disruption buffer. Both the cytoplasmic fraction and the nuclear fraction were separately mixed with 2x lysis/binding solution at room temperature, and 100% ethanol was then added. The sample mixture was added to the collection tube and centrifuged, and the sample mixture was then added to the rescue tube for washing steps. Finally, nuclear and cytoplasmic RNA was eluted with hot elution solution.

### Dual-Luciferase Reporter Assay

To demonstrate direct targeting by hsa_circ_0006867, pmirGLO vectors (Promega, United States), containing the respective wild-type or mutated miR-29a-5p 3′UTR, were cotransfected with 50 nM miR-29a-5p mimics or NC controls in 293T and KDF1 cells. The relative luciferase activity was measured 48 h after transfection, and the firefly fluorescein activity was normalized to the expression of Renilla luciferase in each sample.

### RNA-Binding Protein Immunoprecipitation Assay

A Magna RIP RNA-binding protein immunoprecipitation kit (Millipore, Billerica, MA, United States) was used for RIP analysis. Cultured cells were collected and lysed in RIP lysis buffer. Next, either 5 μg of anti-AGO-2 antibody (ab186733, Abcam, Cambridge, United Kingdom) with the protein of interest or 5 μg of negative anti-IgG antibody was added to protein A/G magnetic beads for 1 h at room temperature. The RIP lysate was then removed and added to each bead-antibody complex in the RIP immunoprecipitation buffer (rotating overnight at 4°C). The beads were mixed with proteinase K buffer and incubated at 55°C for 30 min with vibration. The immunoprecipitated RNA was extracted, and the expression levels of hsa_circ_0006867 and miR-29a-5p were detected by qRT–PCR.

### Statistical Analyses

R software (version 3.6.0) was used to integrate and analyze the microarray data. PRISM software (version 7.0; GraphPad Software, United States) was applied for the other statistical analyses. Student’s t test was used to compare continuous variables among groups. *p* < 0.05 was considered statistically significant.

## Conclusion

In summary, our study delineated comprehensive expression data of circRNAs in keloids and identified 327 dysregulated circRNAs through microarray detection. Several potential circRNA–miRNA–mRNA axes were constructed and may offer promising targets for mechanistic research on keloid formation. Here, we first reported the role of hsa_circRNA_0006867 in promoting the proliferation, migration, and invasion of KDFs, which may serve as a potential biomarker and therapeutic target for keloid prevention and treatment.

## Data Availability

The datasets presented in this study can be found in online repositories. The names of the repository/repositories and accession number(s) can be found below: https://www.ncbi.nlm.nih.gov/, GSE184097.

## Abbreviations

AGO, Argonaute; BP, Biological Process; CC, Cellular Component; circRNAs, Circular RNAs; ceRNA, Competing endogenous RNA; DE, Differentially expressed; ECM, Excessive accumulation of extracellular matrix; GO, Gene Ontology; KCCN3, Small conductance calcium-activated potassium channel protein 3; KDFs, Keloid dermal fibroblasts; KEGG, Kyoto Encyclopedia of Gene and Genomes; LRBA, Lipopolysaccharide-responsive and beige-like anchor protein; MF, Molecular Function; NDFs, Normal dermal fibroblasts; qRT-PCR, Quantitative real-time polymerase chain reaction; RIP, RNA-Binding Protein Immunoprecipitation.

## References

[B1] Bijlard E. Kouwenberg C. Timman R. Hovius S. Busschbach J. Mureau M. (2017). Burden of Keloid Disease: A Cross-Sectional Health-Related Quality of Life Assessment. Acta Derm Venerol 97 (2), 225–229. 10.2340/00015555-2498 27378582

[B2] Chen H. Xu X. Lai L. Huo R. Chen M. (2020). Circ_0008450 Downregulates Runx3 to Promote the Proliferation and Epithelial-Mesenchymal Transition of Human Keratinized Epithelial Cells. Cell Cycle 19 (23), 3303–3316. 10.1080/15384101.2020.1842665 33131417 PMC7751651

[B3] Chen J. Wu Y. Luo X. Jin D. Zhou W. Ju Z. (2021). Circular RNA circRHOBTB3 Represses Metastasis by Regulating the HuR-Mediated mRNA Stability of PTBP1 in Colorectal Cancer. Theranostics 11 (15), 7507–7526. 10.7150/thno.59546 34158864 PMC8210600

[B4] Davies P. Cuttle L. Young A. (2021). A Scoping Review of the Methodology Used in Studies of Genetic Influences on the Development of Keloid or Hypertrophic Scarring in Adults and Children After Acute Wounding. Adv. Wound Care 10 (10), 557–570. 10.1089/wound.2020.1386 PMC8312015 33975469

[B5] Dong Z.-R. Ke A.-W. Li T. Cai J.-B. Yang Y.-f. Zhou W. (2021). CircMEMO1 Modulates the Promoter Methylation and Expression of TCF21 to Regulate Hepatocellular Carcinoma Progression and Sorafenib Treatment Sensitivity. Mol. Cancer 20 (1), 75. 10.1186/s12943-021-01361-3 33985545 PMC8117652

[B6] Gold M. H. Nestor M. S. Berman B. Goldberg D. (2020). Assessing Keloid Recurrence Following Surgical Excision and Radiation. Burns & Trauma 8, 1–7. 10.1093/burnst/tkaa031 PMC7666880 33225004

[B7] Huang C. Ogawa R. (2020). The Vascular Involvement in Soft Tissue Fibrosis-Lessons Learned from Pathological Scarring. Ijms 21 (7), 2542. 10.3390/ijms21072542 PMC7177855 32268503

[B8] Jiang T. Sui D. You D. Yao S. Zhang L. Wang Y. (2018). RETRACTED ARTICLE: MiR-29a-5p Inhibits Proliferation and Invasion and Induces Apoptosis in Endometrial Carcinoma via Targeting TPX2. Cell Cycle 17 (10), 1268–1278. 10.1080/15384101.2018.1475829 29888640 PMC6110586

[B9] Lv W. Ren Y. Hou K. Hu W. Yi Y. Xiong M. (2020). Epigenetic Modification Mechanisms Involved in Keloid: Current Status and prospect. Clin. Epigenet 12 (1), 183. 10.1186/s13148-020-00981-8 PMC7690154 33243301

[B10] Ogawa R. Hsu C. K. (2013). Mechanobiological Dysregulation of the Epidermis and Dermis in Skin Disorders and in Degeneration. J. Cel. Mol. Med. 17 (7), 817–822. 10.1111/jcmm.12060 PMC3822886 23672502

[B11] Pang Q. Wang Y. Xu M. Xu J. Xu S. Shen Y. (2019). MicroRNA-152-5p Inhibits Proliferation and Migration and Promotes Apoptosis by Regulating Expression of Smad3 in Human Keloid Fibroblasts. BMB Rep. 52 (3), 202–207. 10.5483/BMBRep.2019.52.3.278 30638178 PMC6476487

[B12] Potier M. Joulin V. Roger S. Besson P. Jourdan M.-L. Leguennec J.-Y. (2006). Identification of SK3 Channel as a New Mediator of Breast Cancer Cell Migration. Mol. Cancer Ther. 5 (11), 2946–2953. 10.1158/1535-7163.Mct-06-0194 17121942

[B13] Qi X. Zhang D.-H. Wu N. Xiao J.-H. Wang X. Ma W. (2015). ceRNA in Cancer: Possible Functions and Clinical Implications. J. Med. Genet. 52 (10), 710–718. 10.1136/jmedgenet-2015-103334 26358722

[B14] Qu M. Song N. Chai G. Wu X. Liu W. (2013). Pathological Niche Environment Transforms Dermal Stem Cells to Keloid Stem Cells: a Hypothesis of Keloid Formation and Development. Med. Hypotheses 81 (5), 807–812. 10.1016/j.mehy.2013.08.033 24074897

[B15] Ross J. P. Kassir Z. (2014). The Varied Roles of Nuclear Argonaute-Small RNA Complexes and Avenues for Therapy. Mol. Ther. - Nucleic Acids 3, e203. 10.1038/mtna.2014.54 25313622 PMC4217078

[B16] Salmena L. Poliseno L. Tay Y. Kats L. Pandolfi P. P. (2011). A ceRNA Hypothesis: the Rosetta Stone of a Hidden RNA Language? Cell 146 (3), 353–358. 10.1016/j.cell.2011.07.014 21802130 PMC3235919

[B17] Shenton F. Bewick G. S. Banks R. W. (2014). A Study of the Expression of Small Conductance Calcium-Activated Potassium Channels (SK1-3) in Sensory Endings of Muscle Spindles and Lanceolate Endings of Hair Follicles in the Rat. PLoS One 9 (9), e107073. 10.1371/journal.pone.0107073 25191752 PMC4156425

[B18] Shih B. Garside E. McGrouther D. A. Bayat A. (2010). Molecular Dissection of Abnormal Wound Healing Processes Resulting in Keloid Disease. Wound Repair Regen. 18 (2), 139–153. 10.1111/j.1524-475X.2009.00553.x 20002895

[B19] Sinha T. Panigrahi C. Das D. Panda A. (2021). Circular RNA Translation, A Path to Hidden Proteome. Wiley Interdiscip Rev RNA 13 (1), e1685. 10.1002/wrna.1685 34342387 PMC7613019

[B20] Tam O. H. Aravin A. A. Stein P. Girard A. Murchison E. P. Cheloufi S. (2008). Pseudogene-derived Small Interfering RNAs Regulate Gene Expression in Mouse Oocytes. Nature 453 (7194), 534–538. 10.1038/nature06904 18404147 PMC2981145

[B21] Wan H. Zhao S. Zeng Q. Tan Y. Zhang C. Liu L. (2021). CircRNAs in Diabetic Cardiomyopathy. Clinica Chim. Acta 517, 127–132. 10.1016/j.cca.2021.03.001 33711326

[B22] Wang J. Zhang Y. Zhang N. Wang C. Herrler T. Li Q. (2015). An Updated Review of Mechanotransduction in Skin Disorders: Transcriptional Regulators, Ion Channels, and microRNAs. Cell. Mol. Life Sci. 72 (11), 2091–2106. 10.1007/s00018-015-1853-y 25681865 PMC11113187

[B23] Wong V. W. Akaishi S. Longaker M. T. Gurtner G. C. (2011). Pushing Back: Wound Mechanotransduction in Repair and Regeneration. J. Invest. Dermatol. 131 (11), 2186–2196. 10.1038/jid.2011.212 21776006

[B24] Xue M. Zhao R. March L. Jackson C. (2022). Dermal Fibroblast Heterogeneity and Its Contribution to the Skin Repair and Regeneration. Adv. Wound Care 11, 87–107. 10.1089/wound.2020.1287 33607934

[B25] Yan M. Fu L.-L. Nada O. A. Chen L.-M. Gosau M. Smeets R. (2021). Evaluation of the Effects of Human Dental Pulp Stem Cells on the Biological Phenotype of Hypertrophic Keloid Fibroblasts. Cells 10 (7), 1803. 10.3390/cells10071803 34359971 PMC8303871

[B26] Yang Y. Lei W. Jiang S. Ding B. Wang C. Chen Y. (2021). CircRNAs: Decrypting the Novel Targets of Fibrosis and Aging. Ageing Res. Rev. 70, 101390. 10.1016/j.arr.2021.101390 34118443

[B27] Yuan F. L. Sun Z. L. Feng Y. Liu S. Y. Du Y. Yu S. (2019). Epithelial-mesenchymal Transition in the Formation of Hypertrophic Scars and Keloids. J. Cel Physiol 234 (12), 21662–21669. 10.1002/jcp.28830 31106425

[B28] Zhang D. Li B. Zhao M. (2021). Therapeutic Strategies by Regulating Interleukin Family to Suppress Inflammation in Hypertrophic Scar and Keloid. Front. Pharmacol. 12, 667763. 10.3389/fphar.2021.667763 33959031 PMC8093926

[B29] Zhang J.-x. Lu J. Xie H. Wang D.-p. Ni H.-e. Zhu Y. (2019). circHIPK3 Regulates Lung Fibroblast-To-Myofibroblast Transition by Functioning as a Competing Endogenous RNA. Cell Death Dis 10 (3), 182. 10.1038/s41419-019-1430-7 30796204 PMC6385182

[B30] Zhang M. Huang N. Yang X. Luo J. Yan S. Xiao F. (2018). A Novel Protein Encoded by the Circular Form of the SHPRH Gene Suppresses Glioma Tumorigenesis. Oncogene 37 (13), 1805–1814. 10.1038/s41388-017-0019-9 29343848

[B31] Zhao B. Li Z. Qin C. Li T. Wang Y. Cao H. (2021). Mobius Strip in Pancreatic Cancer: Biogenesis, Function and Clinical Significance of Circular RNAs. Cel. Mol. Life Sci. 78, 6201–6213. 10.1007/s00018-021-03908-5 PMC11073466 34342664

