# Supplementary material for: Comprehensive Analysis of Circular RNA Expression in ceRNA Networks and Identification of the Effects of hsa_circ_0006867 in Keloid Dermal Fibroblasts
Source: Front Mol Biosci. 2022 Jan 31;9:800122. doi: 10.3389/fmolb.2022.800122 (PMC8841745; doi:10.3389/fmolb.2022.800122)
Supplement: Supplementary file 1 [file Table1.pdf]

Table S1.The summary of ceRNA network

SeqName	CeNames	CeSymbols	CommonMirnas
hsa_circRNA_103908	ENST00000379328	GATA3	hsa-miR-2682-5p,hsa-miR-449c-5p
hsa_circRNA_103908	ENST00000445964	INPP5D	hsa-miR-4474-3p,hsa-miR-449c-5p
hsa_circRNA_103908	ENST00000223023	WASL	hsa-miR-4474-3p,hsa-miR-449c-5p,hsa-miR-1183
hsa_circRNA_004662	ENST00000524325	TSNARE1	hsa-miR-6811-5p
hsa_circRNA_000479	ENST00000359175	SPTSSB	hsa-miR-4753-3p,hsa-miR-6809-3p
hsa_circRNA_000479	ENST00000287907	HTR5A	hsa-miR-942-5p,hsa-miR-4753-3p,hsa-miR-6809-3p
hsa_circRNA_000479	ENST00000367245	MYCT1	hsa-miR-3925-3p,hsa-miR-4753-3p,hsa-miR-6809-3p
hsa_circRNA_102787	ENST00000303915	ZNF3	hsa-miR-3692-5p,hsa-miR-6071,hsa-miR-6876-3p
hsa_circRNA_000479	ENST00000343457	LRRC66	hsa-miR-4753-3p,hsa-miR-6809-3p
hsa_circRNA_103908	ENST00000391369	AL136115.1	hsa-miR-2682-5p,hsa-miR-449c-5p
hsa_circRNA_000479	ENST00000274569	PCYOX1L	hsa-miR-942-5p,hsa-miR-4753-3p,hsa-miR-6809-3p
hsa_circRNA_102787	ENST00000370017	FNDC7	hsa-miR-3692-5p,hsa-miR-6868-5p
hsa_circRNA_000479	ENST00000641329	OR9K2	hsa-miR-942-5p,hsa-miR-6809-3p
hsa_circRNA_000479	ENST00000621775	USP27X	hsa-miR-942-5p,hsa-miR-6809-3p
hsa_circRNA_004662	ENST00000358278	TPM1	hsa-miR-4520-2-3p
hsa_circRNA_103908	ENST00000622132	ABCB1	hsa-miR-2682-5p,hsa-miR-449c-5p
hsa_circRNA_103908	ENST00000641600	OR8H1	hsa-miR-4289,hsa-miR-449c-5p
hsa_circRNA_103908	ENST00000379554	INSC	hsa-miR-6747-5p,hsa-miR-7110-5p,hsa-miR-449c-5p
hsa_circRNA_000479	ENST00000457167	DNAJC7	hsa-miR-4753-3p,hsa-miR-6809-3p
hsa_circRNA_103908	ENST00000475153	ZNF398	hsa-miR-2682-5p,hsa-miR-449c-5p
hsa_circRNA_103908	ENST00000216373	SOS2	hsa-miR-2682-5p,hsa-miR-449c-5p
hsa_circRNA_103752	ENST00000537866	RSPRY1	hsa-miR-29a-5p
hsa_circRNA_103752	ENST00000392904	IGF1	hsa-miR-1182
hsa_circRNA_103908	ENST00000443824	CTPS2	hsa-miR-2682-5p,hsa-miR-449c-5p
hsa_circRNA_102787	ENST00000477665	PLCXD2	hsa-miR-3692-5p,hsa-miR-6071
hsa_circRNA_000479	ENST00000396331	LILRB1	hsa-miR-4753-3p,hsa-miR-6809-3p
hsa_circRNA_103752	ENST00000380205	IFNA8	hsa-miR-1182
hsa_circRNA_102787	ENST00000332783	ASB7	hsa-miR-3692-5p,hsa-miR-4776-5p,hsa-miR-6071
hsa_circRNA_103908	ENST00000265840	ELMOD1	hsa-miR-2682-5p,hsa-miR-449c-5p
hsa_circRNA_103908	ENST00000255262	NMUR2	hsa-miR-2682-5p,hsa-miR-449c-5p
hsa_circRNA_103908	ENST00000284311	GPR15	hsa-miR-2682-5p,hsa-miR-449c-5p
hsa_circRNA_103908	ENST00000449131	BEST1	hsa-miR-6747-5p,hsa-miR-2682-5p,hsa-miR-4474-3p,hsa-miR-449c-5p
hsa_circRNA_103752	ENST00000296142	RTP3	hsa-miR-29a-5p
hsa_circRNA_103908	ENST00000368990	PLEKHA1	hsa-miR-2682-5p,hsa-miR-449c-5p,hsa-miR-1183
hsa_circRNA_103752	ENST00000379359	RGCC	hsa-miR-29a-5p
hsa_circRNA_000479	ENST00000229812	STK38	hsa-miR-942-5p,hsa-miR-6809-3p
hsa_circRNA_004662	ENST00000261811	CYSTM1	hsa-miR-4520-2-3p
hsa_circRNA_103908	ENST00000445902	VPS52	hsa-miR-2682-5p,hsa-miR-449c-5p
hsa_circRNA_103908	ENST00000210313	PSMD5	hsa-miR-2682-5p,hsa-miR-449c-5p
hsa_circRNA_000479	ENST00000263464	BIRC3	hsa-miR-6809-3p
hsa_circRNA_102787	ENST00000354891	EGFLAM	hsa-miR-3692-5p,hsa-miR-6071
hsa_circRNA_000479	ENST00000306954	GON7	hsa-miR-4753-3p,hsa-miR-146a-3p,hsa-miR-6809-3p,hsa-miR-5189-5p
hsa_circRNA_103908	ENST00000241256	GHSR	hsa-miR-2682-5p,hsa-miR-134-5p,hsa-miR-449c-5p
hsa_circRNA_103908	ENST00000356231	CDAN1	hsa-miR-2682-5p,hsa-miR-449c-5p
hsa_circRNA_000479	ENST00000283296	ADGRF5	hsa-miR-4753-3p,hsa-miR-6809-3p
hsa_circRNA_103908	ENST00000382363	RNF215	hsa-miR-2682-5p,hsa-miR-449c-5p
hsa_circRNA_000479	ENST00000317868	ATG4C	hsa-miR-4753-3p,hsa-miR-146a-3p,hsa-miR-6809-3p
hsa_circRNA_103752	ENST00000361970	CCDC152	hsa-miR-29a-5p,hsa-miR-1182
hsa_circRNA_000479	ENST00000448849	BIVM	hsa-miR-4753-3p,hsa-miR-6809-3p
hsa_circRNA_103908	ENST00000252242	KRT5	hsa-miR-2682-5p,hsa-miR-449c-5p
hsa_circRNA_102787	ENST00000404933	SMS	hsa-miR-3692-5p,hsa-miR-6071,hsa-miR-6876-3p
hsa_circRNA_103908	ENST00000562595	COG8	hsa-miR-2682-5p,hsa-miR-449c-5p,hsa-miR-1183
hsa_circRNA_000479	ENST00000358196	GAD1	hsa-miR-146a-3p,hsa-miR-6809-3p
hsa_circRNA_000479	ENST00000458143	ZNF746	hsa-miR-3925-3p,hsa-miR-6809-3p
hsa_circRNA_103908	ENST00000406427	PNPLA7	hsa-miR-2682-5p,hsa-miR-449c-5p
hsa_circRNA_000479	ENST00000370509	CREG1	hsa-miR-4753-3p,hsa-miR-6809-3p
hsa_circRNA_103908	ENST00000515030	ACSL1	hsa-miR-2682-5p,hsa-miR-449c-5p

hsa_circRNA_000479	ENST00000336378	WASHC2C	hsa-miR-6809-3p
hsa_circRNA_000479	ENST00000277165	FAM120A	hsa-miR-4753-3p,hsa-miR-6809-3p
hsa_circRNA_103908	ENST00000419869	STRAP	hsa-miR-2682-5p,hsa-miR-449c-5p
hsa_circRNA_103908	ENST00000306100	FSTL5	hsa-miR-2682-5p,hsa-miR-449c-5p
hsa_circRNA_103908	ENST00000380122	TXLNG	hsa-miR-589-3p,hsa-miR-6747-5p,hsa-miR-2682-5p,hsa-miR-449c-5p
hsa_circRNA_000479	ENST00000320876	SMCHD1	hsa-miR-4753-3p,hsa-miR-6809-3p
hsa_circRNA_103908	ENST00000511437	FAM173B	hsa-miR-2682-5p,hsa-miR-449c-5p
hsa_circRNA_000479	ENST00000337019	PACRG	hsa-miR-942-5p,hsa-miR-6809-3p
hsa_circRNA_000479	ENST00000412770	PPP6R1	hsa-miR-6809-3p,hsa-miR-5189-5p
hsa_circRNA_000479	ENST00000155926	TRIB2	hsa-miR-942-5p,hsa-miR-6809-3p
hsa_circRNA_000479	ENST00000333870	SPEM2	hsa-miR-6809-3p
hsa_circRNA_102787	ENST00000327813	KREMEN1	hsa-miR-3692-5p,hsa-miR-6071
hsa_circRNA_103908	ENST00000431606	MED19	hsa-miR-2682-5p,hsa-miR-449c-5p
hsa_circRNA_103908	ENST00000225728	MED31	hsa-miR-2682-5p,hsa-miR-449c-5p
hsa_circRNA_103908	ENST00000643399	HK1	hsa-miR-2682-5p,hsa-miR-218-1-3p,hsa-miR-449c-5p
hsa_circRNA_103908	ENST00000634910	HIST1H2BE	hsa-miR-2682-5p,hsa-miR-449c-5p
hsa_circRNA_000479	ENST00000374301	MTFR1L	hsa-miR-942-5p,hsa-miR-4753-3p,hsa-miR-6809-3p,hsa-miR-5189-5p
hsa_circRNA_004662	ENST00000509167	AADAT	hsa-miR-6811-5p
hsa_circRNA_102787	ENST00000229390	SRSF9	hsa-miR-4727-5p,hsa-miR-887-5p,hsa-miR-6878-3p
hsa_circRNA_103752	ENST00000342526	AGMO	hsa-miR-5195-3p,hsa-miR-1182
hsa_circRNA_004662	ENST00000230658	ISL1	hsa-miR-4520-2-3p
hsa_circRNA_004662	ENST00000373517	NAP1L2	hsa-miR-6811-5p
hsa_circRNA_103908	ENST00000261439	TBC1D1	hsa-miR-2682-5p,hsa-miR-4474-3p,hsa-miR-449c-5p
hsa_circRNA_000479	ENST00000257336	BIVM	hsa-miR-4753-3p,hsa-miR-6809-3p
hsa_circRNA_000479	ENST00000377673	FAM216A	hsa-miR-942-5p,hsa-miR-6809-3p
hsa_circRNA_102787	ENST00000308724	AP1G2	hsa-miR-3692-5p
hsa_circRNA_103908	ENST00000450142	PPP1R12A	hsa-miR-589-3p,hsa-miR-2682-5p,hsa-miR-449c-5p
hsa_circRNA_103908	ENST00000370759	GIPC2	hsa-miR-4289,hsa-miR-2682-5p,hsa-miR-449c-5p
hsa_circRNA_103908	ENST00000472487	MYSM1	hsa-miR-589-3p,hsa-miR-2682-5p,hsa-miR-449c-5p
hsa_circRNA_000479	ENST00000542188	TRPM1	hsa-miR-4753-3p,hsa-miR-6809-3p
hsa_circRNA_102787	ENST00000525428	CLNS1A	hsa-miR-3692-5p,hsa-miR-6071
hsa_circRNA_103908	ENST00000265431	CALB1	hsa-miR-2682-5p,hsa-miR-449c-5p
hsa_circRNA_103908	ENST00000377575	HMGN4	hsa-miR-2682-5p,hsa-miR-449c-5p
hsa_circRNA_102787	ENST00000330794	TMEM173	hsa-miR-3692-5p,hsa-miR-6071
hsa_circRNA_000479	ENST00000641926	OR51G2	hsa-miR-942-5p,hsa-miR-6809-3p
hsa_circRNA_103908	ENST00000344922	MIA3	hsa-miR-449c-5p,hsa-miR-1183
hsa_circRNA_103752	ENST00000448599	PHGR1	hsa-miR-4776-3p
hsa_circRNA_103908	ENST00000523189	RANBP17	hsa-miR-2682-5p,hsa-miR-449c-5p
hsa_circRNA_000479	ENST00000482457	ZNF80	hsa-miR-4753-3p,hsa-miR-6809-3p
hsa_circRNA_103752	ENST00000372833	CHCHD1	hsa-miR-1182
hsa_circRNA_103908	ENST00000359865	MTCL1	hsa-miR-2682-5p,hsa-miR-449c-5p
hsa_circRNA_103908	ENST00000300245	AKTIP	hsa-miR-2682-5p,hsa-miR-449c-5p
hsa_circRNA_103908	ENST00000289316	HIST1H2BD	hsa-miR-2682-5p,hsa-miR-4474-3p,hsa-miR-134-5p,hsa-miR-449c-5p
hsa_circRNA_103908	ENST00000569417	MLST8	hsa-miR-2682-5p,hsa-miR-449c-5p
hsa_circRNA_103908	ENST00000279392	HIRIP3	hsa-miR-2682-5p,hsa-miR-449c-5p
hsa_circRNA_000479	ENST00000374855	ALDOB	hsa-miR-4753-3p,hsa-miR-6809-3p
hsa_circRNA_103908	ENST00000581977	IKBKE	hsa-miR-2682-5p,hsa-miR-449c-5p
hsa_circRNA_000479	ENST00000356535	FABP7	hsa-miR-4753-3p,hsa-miR-6809-3p
hsa_circRNA_103908	ENST00000366756	DLL1	hsa-miR-2682-5p,hsa-miR-449c-5p
hsa_circRNA_103908	ENST00000456849	PAPSS2	hsa-miR-589-3p,hsa-miR-449c-5p
hsa_circRNA_103908	ENST00000356033	JPT1	hsa-miR-2682-5p,hsa-miR-449c-5p
hsa_circRNA_103908	ENST00000641432	OR10K1	hsa-miR-2682-5p,hsa-miR-218-1-3p,hsa-miR-449c-5p
hsa_circRNA_103908	ENST00000402399	TCL1A	hsa-miR-2682-5p,hsa-miR-449c-5p
hsa_circRNA_004662	ENST00000520394	TENM2	hsa-miR-4520-2-3p
hsa_circRNA_103908	ENST00000274364	IQGAP2	hsa-miR-2682-5p,hsa-miR-449c-5p
hsa_circRNA_103908	ENST00000273857	CORIN	hsa-miR-6747-5p,hsa-miR-449c-5p
hsa_circRNA_000479	ENST00000262352	SLC1A1	hsa-miR-942-5p,hsa-miR-4753-3p,hsa-miR-6809-3p
hsa_circRNA_103908	ENST00000264935	CEP72	hsa-miR-589-3p,hsa-miR-2682-5p,hsa-miR-449c-5p
hsa_circRNA_102787	ENST00000298815	ARHGAP42	hsa-miR-3692-5p,hsa-miR-6071

hsa_circRNA_000479	ENST00000207457	TEKT2	hsa-miR-6809-3p
hsa_circRNA_102787	ENST00000393997	ELMO3	hsa-miR-3692-5p
hsa_circRNA_000479	ENST00000276571	CRH	hsa-miR-4753-3p,hsa-miR-6809-3p
hsa_circRNA_000479	ENST00000378487	CLLU1OS	hsa-miR-942-5p,hsa-miR-4753-3p,hsa-miR-6809-3p
hsa_circRNA_103908	ENST00000355999	STK39	hsa-miR-6747-5p,hsa-miR-2682-5p,hsa-miR-7110-5p,hsa-miR-449c-5p
hsa_circRNA_000479	ENST00000373212	SH3BGRL	hsa-miR-4753-3p,hsa-miR-6809-3p
hsa_circRNA_102787	ENST00000301671	GHDC	hsa-miR-3692-5p,hsa-miR-887-5p
hsa_circRNA_004662	ENST00000229135	IFNG	hsa-miR-6811-5p
hsa_circRNA_004662	ENST00000355530	SPNS3	hsa-miR-6811-5p
hsa_circRNA_103908	ENST00000331923	PAXBP1	hsa-miR-4474-3p,hsa-miR-449c-5p
hsa_circRNA_103752	ENST00000392167	PNLDC1	hsa-miR-29a-5p
hsa_circRNA_103752	ENST00000371218	FGGY	hsa-miR-1182
hsa_circRNA_000479	ENST00000231121	HAND1	hsa-miR-4753-3p,hsa-miR-6809-3p
hsa_circRNA_103908	ENST00000262103	EFCAB1	hsa-miR-2682-5p,hsa-miR-449c-5p
hsa_circRNA_103908	ENST00000337573	EBAG9	hsa-miR-2682-5p,hsa-miR-449c-5p
hsa_circRNA_103752	ENST00000370784	FAM122C	hsa-miR-29a-5p
hsa_circRNA_103752	ENST00000419673	ARHGAP28	hsa-miR-6082,hsa-miR-5195-3p,hsa-miR-1182
hsa_circRNA_000479	ENST00000409911	LRRTM4	hsa-miR-4753-3p,hsa-miR-6809-3p
hsa_circRNA_103908	ENST00000329773	MRGPRX2	hsa-miR-2682-5p,hsa-miR-449c-5p
hsa_circRNA_000479	ENST00000266744	ASCL1	hsa-miR-4753-3p,hsa-miR-6809-3p
hsa_circRNA_103908	ENST00000405666	ESRRA	hsa-miR-2682-5p,hsa-miR-449c-5p
hsa_circRNA_102787	ENST00000526438	DEFB134	hsa-miR-3692-5p,hsa-miR-6071,hsa-miR-6878-3p
hsa_circRNA_103752	ENST00000562767	P11-315D16.	hsa-miR-4776-3p
hsa_circRNA_103908	ENST00000452236	ZBED9	hsa-miR-449c-5p,hsa-miR-1183
hsa_circRNA_103752	ENST00000282605	JADE2	hsa-miR-1182
hsa_circRNA_103908	ENST00000398458	TMPRSS12	hsa-miR-449c-5p
hsa_circRNA_000479	ENST00000367500	SWT1	hsa-miR-4753-3p,hsa-miR-6809-3p
hsa_circRNA_103908	ENST00000360256	F8	hsa-miR-2682-5p,hsa-miR-7110-5p,hsa-miR-449c-5p
hsa_circRNA_103908	ENST00000518111	STMN2	hsa-miR-2682-5p,hsa-miR-449c-5p
hsa_circRNA_103752	ENST00000035383	LRRC7	hsa-miR-4776-3p
hsa_circRNA_000479	ENST00000332127	ZC3H11A	hsa-miR-4753-3p,hsa-miR-6809-3p
hsa_circRNA_103908	ENST00000324907	GRHL1	hsa-miR-2682-5p,hsa-miR-449c-5p
hsa_circRNA_103908	ENST00000305883	KLF11	hsa-miR-2682-5p,hsa-miR-449c-5p
hsa_circRNA_000479	ENST00000641470	OR6F1	hsa-miR-6809-3p
hsa_circRNA_000479	ENST00000381095	NLGN4X	hsa-miR-942-5p,hsa-miR-4753-3p,hsa-miR-6809-3p
hsa_circRNA_103752	ENST00000524142	ATOX1	hsa-miR-1182
hsa_circRNA_000479	ENST00000315184	FAM71F1	hsa-miR-942-5p,hsa-miR-6809-3p
hsa_circRNA_103908	ENST00000261783	ARG2	hsa-miR-2682-5p,hsa-miR-449c-5p
hsa_circRNA_000479	ENST00000354185	DDX21	hsa-miR-942-5p,hsa-miR-4753-3p,hsa-miR-6809-3p
hsa_circRNA_004662	ENST00000368904	CUZD1	hsa-miR-6811-5p
hsa_circRNA_000479	ENST00000314664	WASHC2A	hsa-miR-6809-3p
hsa_circRNA_103908	ENST00000380986	FKBP1B	hsa-miR-2682-5p,hsa-miR-449c-5p
hsa_circRNA_103908	ENST00000361272	CNOT7	hsa-miR-2682-5p,hsa-miR-4474-3p,hsa-miR-449c-5p
hsa_circRNA_103752	ENST00000325144	ZBTB2	hsa-miR-4776-3p,hsa-miR-29a-5p
hsa_circRNA_103752	ENST00000359429	RPE	hsa-miR-4776-3p,hsa-miR-1255b-2-3p
hsa_circRNA_004662	HBMT00001081968	'G000000723	hsa-miR-4520-2-3p
hsa_circRNA_103908	ENST00000498707	ADAMTS9	hsa-miR-2682-5p,hsa-miR-449c-5p
hsa_circRNA_103908	ENST00000620048	DENND1B	hsa-miR-2682-5p,hsa-miR-4474-3p,hsa-miR-449c-5p,hsa-miR-1183
hsa_circRNA_103908	ENST00000375006	PFKFB1	hsa-miR-2682-5p,hsa-miR-449c-5p
hsa_circRNA_103908	ENST00000256398	ELP3	hsa-miR-6747-5p,hsa-miR-2682-5p,hsa-miR-449c-5p
hsa_circRNA_000479	ENST00000316073	RPTN	hsa-miR-4753-3p,hsa-miR-6809-3p
hsa_circRNA_103908	ENST00000421852	TMEM41A	hsa-miR-589-3p,hsa-miR-2682-5p,hsa-miR-449c-5p
hsa_circRNA_000479	ENST00000271532	FCRL4	hsa-miR-942-5p,hsa-miR-3925-3p,hsa-miR-4753-3p,hsa-miR-6809-3p
hsa_circRNA_103752	ENST00000601759	AC110084.1	hsa-miR-29a-5p
hsa_circRNA_000479	ENST00000262053	ATF1	hsa-miR-942-5p,hsa-miR-6809-3p
hsa_circRNA_000479	ENST00000307897	ZWILCH	hsa-miR-4753-3p,hsa-miR-6809-3p
hsa_circRNA_103752	ENST00000407426	WDR43	hsa-miR-5195-3p,hsa-miR-4776-3p
hsa_circRNA_103908	ENST00000379638	REEP5	hsa-miR-2682-5p,hsa-miR-449c-5p
hsa_circRNA_103908	ENST00000340369	XIRP1	hsa-miR-2682-5p,hsa-miR-449c-5p

hsa_circRNA_102787	ENST00000307450	MORN4	hsa-miR-3692-5p,hsa-miR-6071,hsa-miR-7150
hsa_circRNA_000479	ENST00000233638	TLX2	hsa-miR-3925-3p,hsa-miR-6809-3p
hsa_circRNA_103908	ENST00000555993	MAP3K9	hsa-miR-2682-5p,hsa-miR-449c-5p
hsa_circRNA_000479	ENST00000521508	ALG11	hsa-miR-942-5p,hsa-miR-4753-3p,hsa-miR-6809-3p
hsa_circRNA_103908	ENST00000517294	CCDC160	hsa-miR-2682-5p,hsa-miR-449c-5p
hsa_circRNA_000479	ENST00000338008	ZFYVE16	hsa-miR-942-5p,hsa-miR-4753-3p,hsa-miR-6809-3p
hsa_circRNA_000479	ENST00000319217	MPDZ	hsa-miR-942-5p,hsa-miR-4753-3p,hsa-miR-6809-3p
hsa_circRNA_103908	ENST00000238738	RHOQ	hsa-miR-2682-5p,hsa-miR-4474-3p,hsa-miR-449c-5p
hsa_circRNA_102787	ENST00000263246	PACSIN2	hsa-miR-3692-5p,hsa-miR-6071
hsa_circRNA_000479	ENST00000632372	TEX13D	hsa-miR-4753-3p,hsa-miR-6809-3p
hsa_circRNA_103908	ENST00000421367	ERLIN1	hsa-miR-2682-5p,hsa-miR-4474-3p,hsa-miR-449c-5p
hsa_circRNA_102787	ENST00000414429	ZSCAN31	hsa-miR-3692-5p,hsa-miR-4776-5p
hsa_circRNA_103908	ENST00000221200	KCTD9	hsa-miR-2682-5p,hsa-miR-449c-5p
hsa_circRNA_103908	ENST00000276893	UHRF2	hsa-miR-2682-5p,hsa-miR-449c-5p
hsa_circRNA_004662	ENCT00000214852	G000000409	hsa-miR-4520-2-3p
hsa_circRNA_103908	ENST00000334828	PGAM1	hsa-miR-2682-5p,hsa-miR-134-5p,hsa-miR-449c-5p
hsa_circRNA_000479	ENST00000393539	ZNF23	hsa-miR-4753-3p,hsa-miR-6809-3p
hsa_circRNA_102787	ENST00000340650	NHLRC1	hsa-miR-4727-5p,hsa-miR-887-5p
hsa_circRNA_103908	ENST00000294484	DISP3	hsa-miR-2682-5p,hsa-miR-449c-5p
hsa_circRNA_102787	ENST00000350889	STMN4	hsa-miR-3692-5p,hsa-miR-4727-5p,hsa-miR-6876-3p
hsa_circRNA_103752	ENST00000502492	SNF8	hsa-miR-4776-3p,hsa-miR-1182
hsa_circRNA_102787	ENST00000359428	NUP214	hsa-miR-3692-5p,hsa-miR-6876-3p
hsa_circRNA_000479	ENST00000367604	HIVEP2	hsa-miR-4753-3p,hsa-miR-6809-3p
hsa_circRNA_103908	ENST00000359440	TMC3	hsa-miR-2682-5p,hsa-miR-449c-5p
hsa_circRNA_103908	ENST00000264255	TXNDC9	hsa-miR-2682-5p,hsa-miR-449c-5p
hsa_circRNA_102787	ENST00000217131	CTSZ	hsa-miR-3692-5p,hsa-miR-6071
hsa_circRNA_102787	ENST00000341170	TSTD2	hsa-miR-3692-5p,hsa-miR-4727-5p,hsa-miR-887-5p,hsa-miR-7150
hsa_circRNA_103908	ENST00000354625	RPS6KL1	hsa-miR-2682-5p,hsa-miR-7110-5p,hsa-miR-449c-5p
hsa_circRNA_000479	ENST00000246841	FLRT1	hsa-miR-6809-3p,hsa-miR-5189-5p
hsa_circRNA_000479	ENST00000641027	OR1L8	hsa-miR-6809-3p
hsa_circRNA_102787	ENST00000431843	SIGIRR	hsa-miR-3692-5p,hsa-miR-7150
hsa_circRNA_000479	ENST00000382611	IRX2	hsa-miR-942-5p,hsa-miR-4753-3p,hsa-miR-6809-3p
hsa_circRNA_103908	ENST00000305620	KRT74	hsa-miR-2682-5p,hsa-miR-134-5p,hsa-miR-7110-5p,hsa-miR-449c-5p
hsa_circRNA_000479	ENST00000429001	LARP4	hsa-miR-146a-3p,hsa-miR-6809-3p
hsa_circRNA_004662	ENST00000357716	GRM7	hsa-miR-4520-2-3p
hsa_circRNA_000479	ENST00000389834	TMEM87A	hsa-miR-4753-3p,hsa-miR-6809-3p
