# Supplementary material for: Comprehensive Analysis of Circular RNA Expression in ceRNA Networks and Identification of the Effects of hsa_circ_0006867 in Keloid Dermal Fibroblasts
Source: Front Mol Biosci. 2022 Jan 31;9:800122. doi: 10.3389/fmolb.2022.800122 (PMC8841745; doi:10.3389/fmolb.2022.800122)

Figure S1. Pathways map associated with up-regulated circRNAs. Pathway analysis showed “Tight Junction" was the most affected pathway related to up-regulated circRNAs. Orange marked nodes were associated with up-regulated or only whole dataset genes.


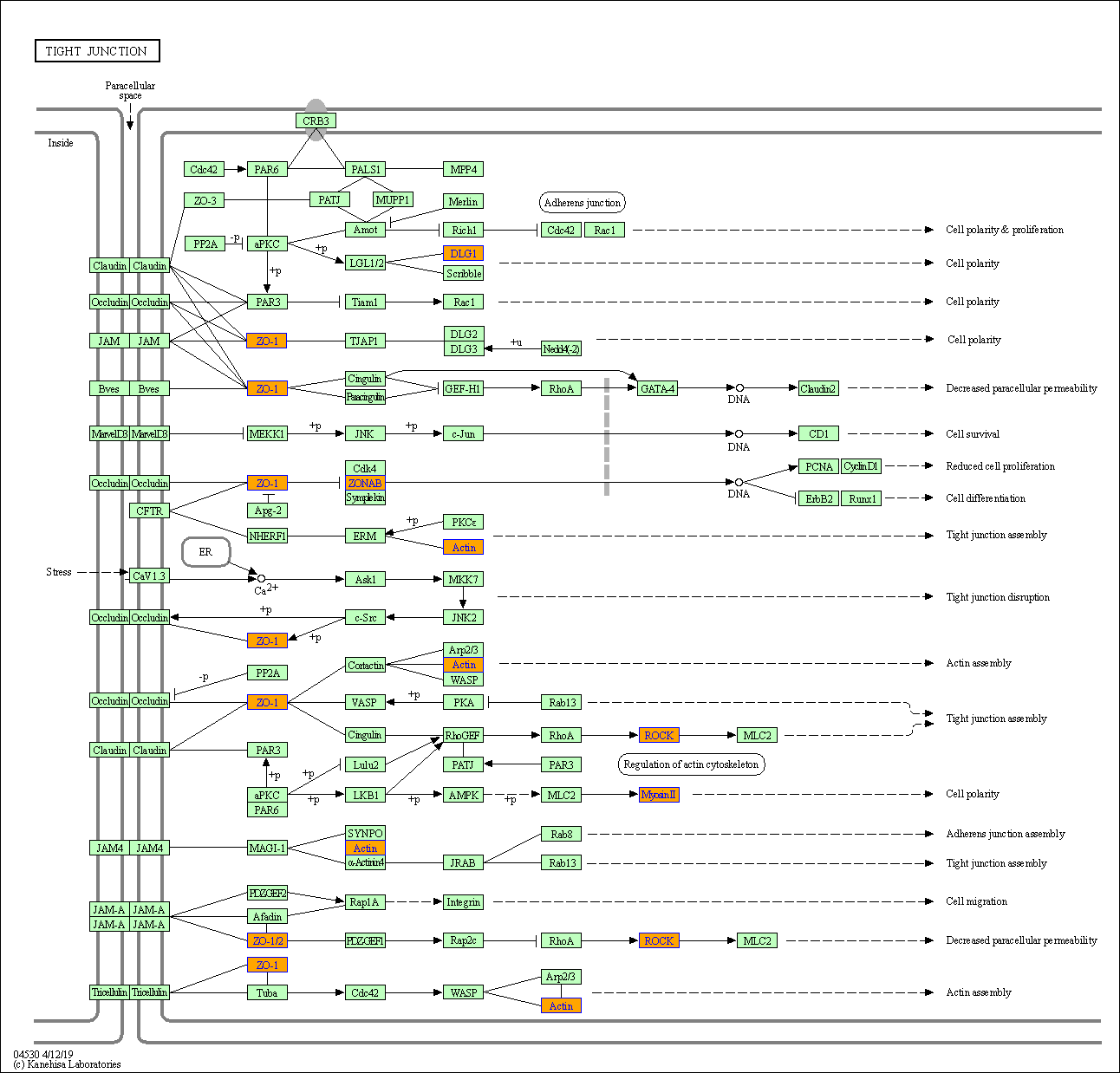


Figure S2. Pathways map associated with down-regulated circRNAs. Pathway analysis showed “Axon Guidance" was the most affected pathway related to down-regulated circRNAs. Yellow marked nodes were associated with down-regulated genes, green nodes have no significance.


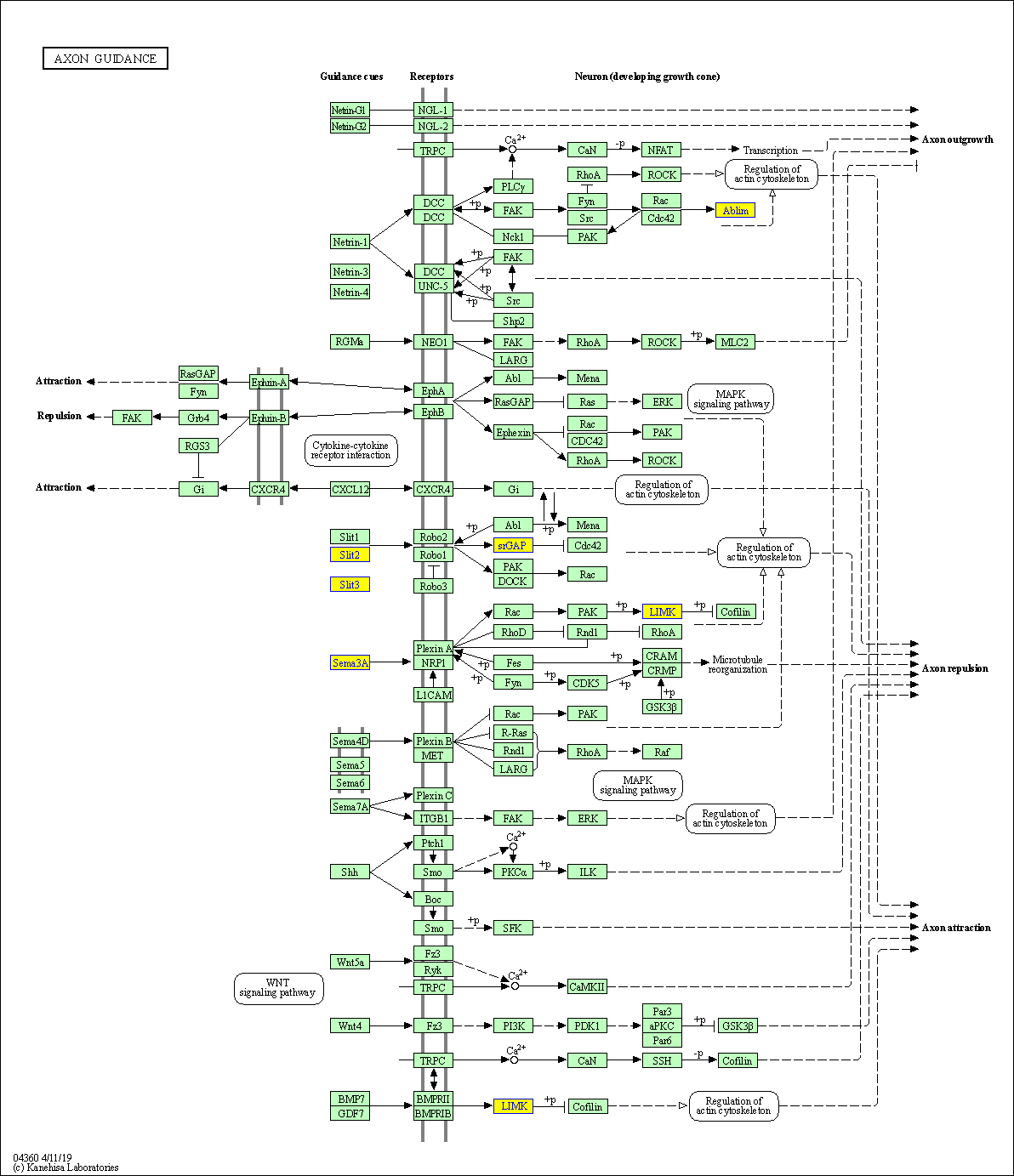

Supplement: Supplementary file 2 [file DataSheet1.DOCX]
